# Hierarchical Assembly of Single-Stranded RNA

**DOI:** 10.1021/acs.jctc.3c01049

**Published:** 2024-02-16

**Authors:** Lisa M. Pietrek, Lukas S. Stelzl, Gerhard Hummer

**Affiliations:** † Department of Theoretical Biophysics, Max Planck Institute of Biophysics, Max-von-Laue-Straße 3, 60438 Frankfurt am Main, Germany; ‡ Faculty of Biology, Johannes Gutenberg University Mainz, Gresemundweg 2, 55128 Mainz, Germany; § KOMET 1, Institute of Physics, Johannes Gutenberg University Mainz, 55099 Mainz, Germany; ∥ Institute of Molecular Biology (IMB), 55128 Mainz, Germany; ⊥ Institute for Biophysics, Goethe University, Max-von-Laue-Straße 9, 60438 Frankfurt am Main, Germany

## Abstract

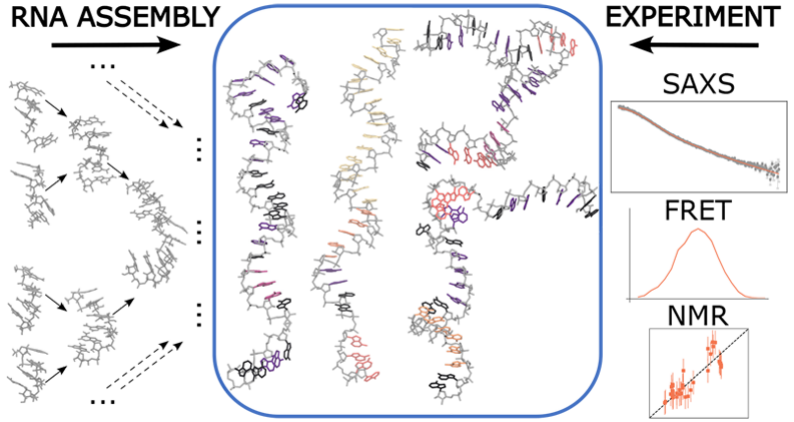

Single-stranded RNA (ssRNA) plays a major role in the flow of genetic information–most notably, in the form of messenger RNA (mRNA)–and in the regulation of biological processes. The highly dynamic nature of chains of unpaired nucleobases challenges structural characterizations of ssRNA by experiments or molecular dynamics (MD) simulations alike. Here, we use hierarchical chain growth (HCG) to construct ensembles of ssRNA chains. HCG assembles the structures of protein and nucleic acid chains from fragment libraries created by MD simulations. Applied to homo- and heteropolymeric ssRNAs of different lengths, we find that HCG produces structural ensembles that overall are in good agreement with diverse experiments, including nuclear magnetic resonance (NMR), small-angle X-ray scattering (SAXS), and single-molecule Förster resonance energy transfer (FRET). The agreement can be further improved by ensemble refinement using Bayesian inference of ensembles (BioEn). HCG can also be used to assemble RNA structures that combine base-paired and base-unpaired regions, as illustrated for the 5′ untranslated region (UTR) of SARS-CoV-2 RNA.

## 1 Introduction

Single-stranded RNAs (ssRNAs) play important roles in many cellular processes, in particular, in the transmission of genetic information in the form of messenger RNA (mRNA). Noncoding stretches in mRNA or fully noncoding ssRNAs have key roles in the regulation of transcription and translation,^1^ e.g., by acting as riboswitches^2^ or by regulating the nuclear export of mRNA, and its stability and translation via polyadenylation.^3^ In solution, ssRNAs can remain dynamically fully flexible and unstructured, transiently adopt secondary structures with paired bases, or form more stable secondary structures in complex with a binding partner.^4,5^ mRNA in rapidly dividing cells was found to be substantially less structured than in vitro.^6^ Therapeutics based on mRNA have long been explored,^7^ which has recently led to the development of vaccines based on mRNA. In addition, nonbase-paired regions in RNA have emerged as promising drug targets.^8^

To improve our understanding of ssRNA and their functional mechanisms, we need to characterize their structural and dynamical features. However, experimentally investigating disordered ssRNA remains a challenging task. Nuclear magnetic resonance (NMR) techniques provide powerful tools to investigate local structure and dynamics with high-resolution in short disordered stretches of ssRNA shifts.^9−15^ Small-angle X-ray scattering (SAXS) studies^16−18^ or Förster resonance energy transfer (FRET) techniques^16,19−21^ yield insight into the global structure of flexible biomolecules. The negatively charged ssRNA molecules have been shown to be strongly dependent on environmental buffer conditions, including ion concentration and type,^11,16−18^ an effect seen also in molecular dynamics (MD) simulations.^9,21^

Structural ensembles of ssRNA that capture the heterogeneity of these highly dynamic systems in atomic detail help the interpretation of data from experiments. Most experiments report ensemble averages. Such ensembles can, in principle, be obtained by performing MD simulations. However, MD simulations suffer from inaccuracies in the available force fields.^22,23^ For RNA, special care has to be taken in setting the buffer conditions and choosing the ion force field parameters.^24,25^ For biopolymers, small systematic errors in, say, backbone torsion potentials add up and result in major structural imbalances.^26^ Inaccuracies in the energetics are amplified by the broad and shallow energy landscape of flexible biomolecules,^13,21,27,28^ which requires extensive sampling. The sampling of ssRNA structural ensembles by MD simulations thus suffers both from systematic uncertainties due to inaccuracies in the force field and from statistical uncertainties due to the slow structural dynamics.

Fragment assembly is a promising approach to model RNA 3D structures. In early applications of RNA fragment assembly, Das et al. used FARFAR, a Rosetta-like fragment assembly approach to model noncanonical double-stranded (dsRNA) structure with atomistic detail.^29^ Their fragment structures were drawn from a library based on RNA structure in the large ribosomal subunit.^29^ The more recent FARFAR2 approach^30^ has been used to generate ensembles of short ssRNA polymers.^14^ Chojnowski et al. developed a method to model 3D structures of short RNA polymers featuring base-paired strands as well as unpaired strands involved in loops by assembling RNA fragments from the PDB with the option to include experimental restraints.^31^

Ensembles of flexible biopolymers can be improved by integrating available experimental data. Approaches such as Bayesian/Maximum Entropy (BME)^32−35^ and Bayesian inference^36−40^ have been shown to work well in applications to ensembles of disordered biomolecules. For instance, Bottaro and co-workers refined tetrameric fragments according to NMR data using a BME approach, to improve their structural ensembles obtained via MD simulation, resulting in a more accurate description of the thermodynamic states.^13^ In another example, Bergonzo et al. showed that a BME approach helped to improve conformational ensembles of a heteropolymeric oligonucleotide by integrating NMR and SAXS experimental data.^14^ Alternatively, integration of experimental information can help to build models of observed molecules.^18,41,42^

In previous work, we have introduced the hierarchical chain growth (HCG) method for disordered proteins.^28,40,43^ We found that HCG is a robust approach well suited to efficiently growing broad structural ensembles of disordered proteins with atomic detail that are consistent with experimental findings. Here, we adapted HCG to model structural ensembles of disordered ssRNA. We focus on systems for which experimental data are available as reference:^14,18,21^ homopolymeric adenosine monophosphate multimers (rA_n_ with *n* = 19, 30), homopolymeric uridine monophosphate 30mer (rU_30_), and the short disordered heteropolymeric ssRNA rUCAAUC. We implemented ssRNA fragment assembly in the form of a Monte Carlo chain growth algorithm, which we then used to assemble structural ensembles of conformations with atomic detail. We validated the modeled ensembles against diverse experimental data and could establish good agreement on average without refinement. We further improved the agreement with experimental observations by integrating experimental data using Bayesian inference of ensembles (BioEn) as a gentle ensemble refinement method.^37^ As a proof of principle, we demonstrate that ssRNA chains grown with HCG can be combined with models of dsRNA, paving the way toward modeling short unstructured linkers, terminal untranslated regions (UTRs), or loops.

## 2 Methods

### 2.1 MD Fragment Library

For poly adenine (A) RNA, an rA_4_ tetramer was modeled using the AMBER suite of programs.^44^ The oxygen atoms of the terminal ribose groups at the 5′ and 3′ ends were protonated (Figure S1). For heteropolymeric ssRNA, we used heterotetrameric fragments rGXYZ. The nucleotide at the 5′ position was fixed as guanine (G). We chose guanine as the headgroup, first, to mimic the interior of the ssRNA by providing a purine platform for stacking and, second, to facilitate the alignment with a relatively large base. For the following three nucleotides “XYZ”, we used all 4^3^ = 64 combinations of G, A, cytosine (C), and uracil (U). Each RNA fragment was placed in a dodecahedral box and solvated in TIP4P-D water^45^ with 150 mM NaCl.^46^ Charge neutrality was established with excess sodium ions. On average, the resulting systems contained about 6600 atoms in total. The RNA fragments were modeled with the DESRES^23^ force field. We thus performed the fragment MD simulations using the same force field, water model, and ion parameters as described before.^21^

MD simulations were performed with GROMACS/2018.8.^47^ Bonds including hydrogen atoms were constrained using the P-LINCS algorithm.^48^ To maintain the pressure at a constant value of 1 bar, the Parrinello–Rahman barostat^49^ was used. The cutoff distances for van der Waals and real-space electrostatic interactions were set to 1.2 nm. Electrostatic interactions were calculated using the particle mesh Ewald method^50^ with the Fourier spacing set to 0.16 nm. The system was first energy minimized, followed by 400 ps of MD equilibration. The production REMD simulation was run in the NPT ensemble for 100 ns, with structures saved every 10 ps. For all tetramer fragments, we used 25 replicas that collectively spanned a temperature range of 300–431 K, as calculated using the algorithm by Patriksson and van der Spoel.^51^ For each system, 10000 different structures collected at equally spaced time points from the replica simulated at 300 K were used for the respective fragment library.

### 2.2 Hierarchical Chain Growth

We adapted HCG^40,43^ to grow full-length models of disordered homo- and heteropolymeric ssRNA chains from MD rA_4_ and rGXYZ fragments. HCG was previously implemented and validated to model extensive ensembles of intrinsically disordered proteins (IDPs), displaying average properties that are in line with experimental observables.^28,40,43^ HCG performs fragment assembly; i.e., a pool of fragment structures is combined at random into long polymers. The structural alignment of individual fragments and the rejection of poorly aligned or sterically clashing fragment pairs are critical for the quality of the resulting ensembles in terms of both local and global structural properties. We note that besides the root-mean-square distance (RMSD) alignment criterion and the steric exclusion we did not include any kind of attractive or repulsive interfragment interaction during the assembly. Thus, only intrafragment electrostatic interactions are considered in HCG, as sampled in the fragment MD simulations. However, we integrate experimental data on a global level to account for possible discrepancies in assembled polymers.

**Figure 1:**
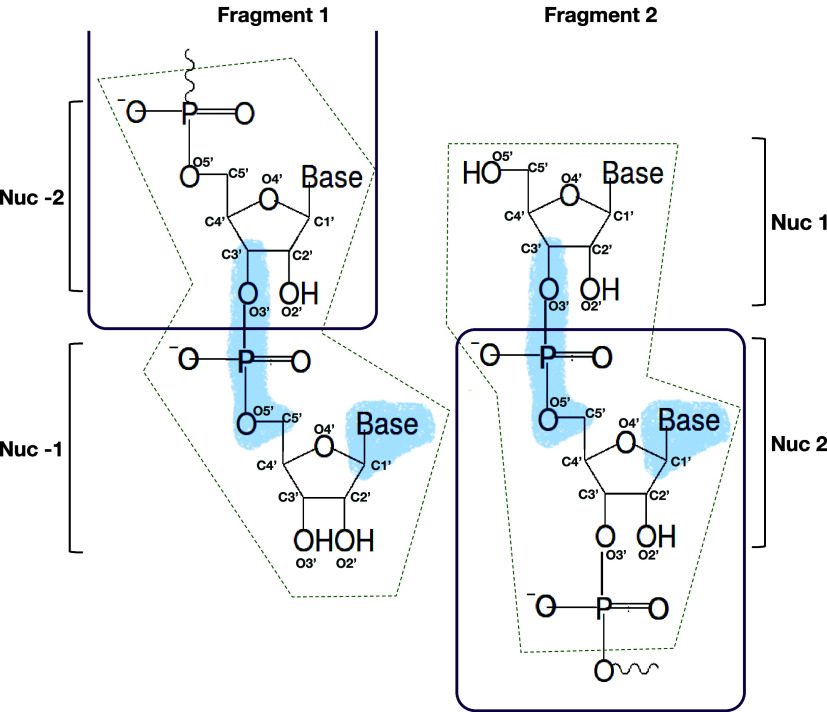
Fragment alignment. Aligned heavy atoms are highlighted by blue shading. Heavy atoms within the dashed dark green boxes were excluded from the clash search. The purple lines indicate the regions of the two fragments that are included in the assembled chain.

We used a fragment alignment strategy that not only focuses on the nucleic acid backbone but also accounts for the position of the base. For two conformations of fragments adjacent in sequence and drawn at random from the respective pool, we performed a rigid-body superimposition of the O3′ atom, phosphate atom, and O5′ atom connecting nucleotides −2 and −1 in fragment 1 and nucleotides 1 and 2 in fragment 2, and all atoms of the nucleobase as well as the C1 atom of nucleotide −1 in fragment 1 and of nucleotide 2 in fragment 2 (Figure 1, light blue shaded area). For a successful alignment, we required the RMSD of the superimposed atoms to be below a given threshold, RMSD < 0.64 Å. In the superimposition, we doubled the weights of the aligned backbone atoms relative to the aligned atoms of the nucleobase to produce atom distances within the expected range.^52^

Alignment was followed by a search for steric clashes, defined as heavy atom distances below a cutoff of 2 Å. Note that we did not consider hydrogen atoms in clash detection. Atoms in the fragment-overlap region were excluded from the heavy atom clash search (Figure 1, dark green dashed boxes). Any steric clash resulted in the rejection of the fragment pair. Otherwise, the two fragments were merged. In merged fragments, nucleotide −1 from the first and nucleotide 1 from the second fragment were removed. In this way, the assembled chain featured only nucleotides sampled at the second and third positions (X and Y) of the rGXYZ fragments. The terminal nucleotides (G and Z) were treated as capping groups. We repeated this procedure in each hierarchical level of HCG until we reached the full-length sequence. For each polymer investigated in the present work, we grew ensembles with 10000 members. The RNA structure libraries (i.e., the MD fragment library as well as exemplary structures from the HCG ensembles discussed in this work) are available at https://zenodo.org/record/8369324. The HCG code to assemble ssRNA to the hierarchical chain growth is available at the GitHub repository https://github.com/bio-phys/hierarchical-chain-growth/.

**Figure 2:**
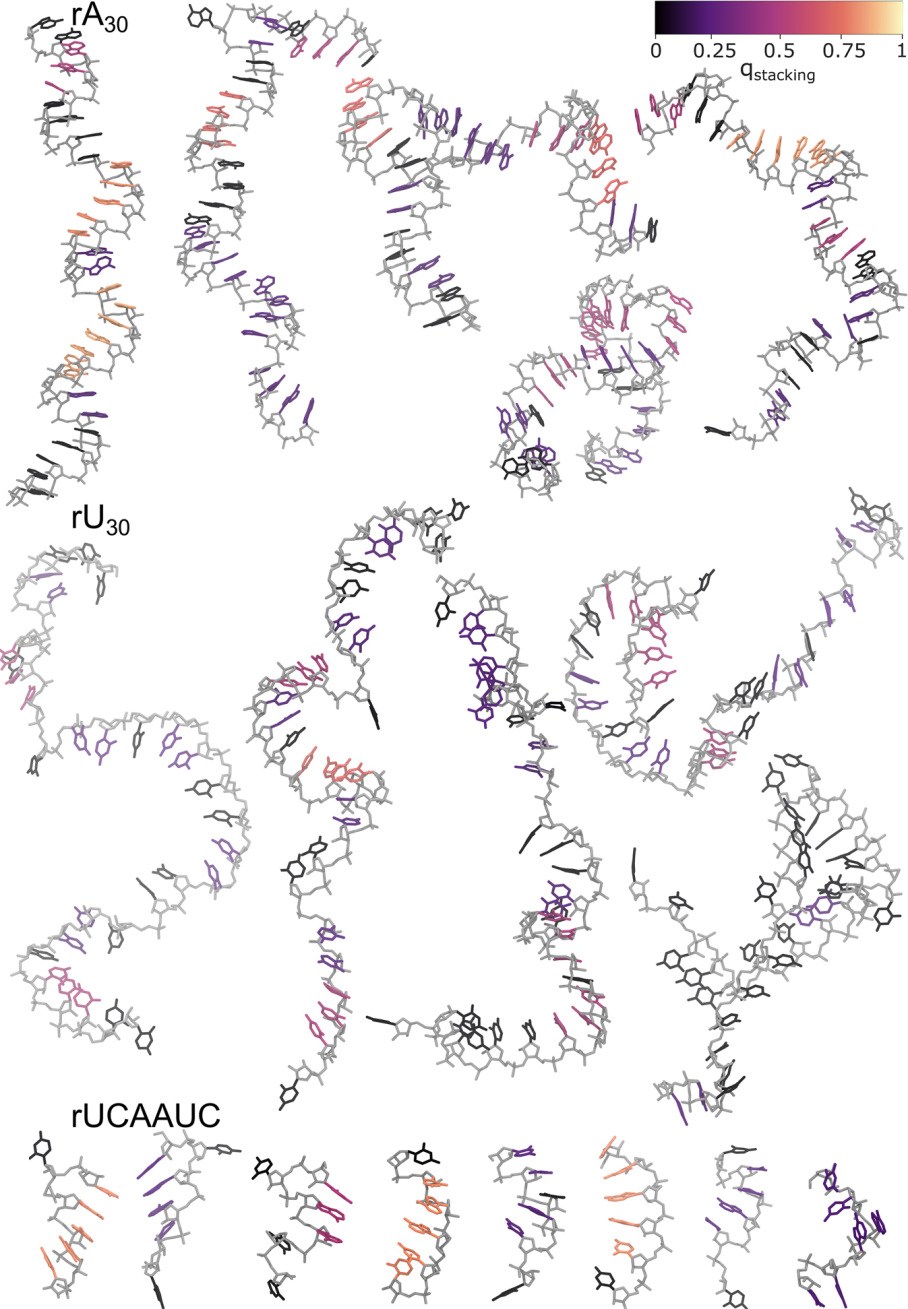
Snapshots of ssRNA polymers grown with HCG. Representative renders of structures drawn at random from ensembles of (top) rA_30_, (center) rU_30_, and (bottom) rUCAAUC sampled by fragment assembly. The nucleic backbone is shown in light gray, and nucleobases are colored according to their base stacking factor^55^*q*_stacking_ (top: color code). Hydrogen atoms are omitted for clarity.

We note that the ribose atoms are not included in the superimposition. We found that by not enforcing the sugar pucker configuration, we increased the diversity of grown structures and benefitted from diverse sugar pucker configurations sampled in fragment MD simulations. By including the nucleobase in the alignment, we improved the configuration of stacked bases, which is important to produce reasonable stacking also for longer sequences (Figure 2). The extent of base stacking in the assembled structures will to a significant degree be predetermined by the fragment library entering HCG and thus the MD simulation force field used to create the library.^11^

### 2.3 Modeling the 5′ UTR of SARS-CoV-2 RNA

To build a structural ensemble of the 5′ UTR of SARS-CoV-2 RNA by HCG, we combined fragments for the ssRNA segments with structural models for the stem loops using the secondary structure as input. The conformations of the structured stem-loop regions were randomly drawn from libraries filled with structures from MD trajectories published previously.^53^ The connected disordered regions were grown using HCG as described above according to the sequence in ref (53). For the assembly, the same scheme as implemented in HCG was used. In particular, the adjacent regions (fragments) were assembled in a hierarchical manner in subsequent levels. In the final level, the full-length model was assembled with a total of 233 nucleotides (sequence and secondary structure in Supplementary Figure S2). For the heavy atom superimposition, we set the RMSD cutoff to 1 Å, and the clash radius was kept at 2 Å. We grew only a small ensemble of 50 full-length chains. We note that the region spanned by nucleotides 162–200 was predicted to be structured and a part of stem-loop SL5.^54^ However, to our knowledge for this region, there has been no structure solved so far. Therefore, we here modeled this region as a single-stranded region with HCG.

### 2.4 Mapping of FRET Labels

HCG is naturally suited to the inclusion of molecular labels, such as covalently attached fluorophores. To build a pool of dye-labeled rA_4_ fragments, we used an MD library for the dyes Alexa Fluor 594 and Alexa Fluor 488 attached to dideoxyadenosinemonophosphate (dA_2_). The use of dA_2_-dye fragments to model fluorophores attached to both DNA and RNA chains has been validated by Grotz et al.^21^ A random structure was drawn from the pool of rA_4_ fragments and from the Alexa Fluor 594 or Alexa Fluor 488 MD library, to either label the 5′ or 3′ end, respectively, of the rA_4_ fragments. We performed a rigid body alignment of heavy atoms of the sugar moiety and nucleobase from the terminal nucleotides. In particular, to attach Alexa Fluor 594 to rA_4_, we aligned the respective atoms from the terminal nucleotide at the 5′ end of the rA_4_ fragment with the respective atoms from the terminal nucleotide at the 3′ end from the dA_2_-dye fragment. For Alexa Fluor 488, the same alignment was performed but at the 3′ end of the rA_4_ fragment and at the 5′ end from the dA_2_-dye fragment. The RMSD cutoff for heavy atom distances was set to 0.8 Å. If the RMSD value was below the cutoff, we searched for clashing heavy atoms within a pair distance of 2.0 Å. If no clashing atoms were detected, then the dye molecules and the rA_4_ fragment were assembled such that all atoms from the dA_2_ fragment and terminal oxygens of rA_4_ were excluded. In this way, we sampled a library of the FRET dyes mapped onto rA_4_ fragments with 10000 conformations for each fluorophore, Alexa Fluor 594 and Alexa Fluor 488.

The fragment libraries used here for the fluorescent dyes contain only a short nucleic acid segment. Attractive interactions between dyes and nucleic acids are thus limited to the terminal bases. With more distant bases, only steric interactions are considered. However, MD simulations can result in excessive sticking of the dye to the nucleic acid with some force fields.^21^ By contrast, experimentalists tend to exclude dyes that stick to the attached RNA or proteins, based on measurements for instance of the fluorescence anisotropy decay.^21,40^ The fragment-based approach to modeling dyes taken here and by Grotz et al.^21^ takes advantage of this strategy, being aimed at the modeling of experiments with nonsticky dyes.

### 2.5 Ensemble Reweighting Using BioEn

We refined the HCG ensembles of the ssRNA polymers investigated here against experimental SAXS or single-molecule FRET data by reweighting using BioEn.^37,38^ We used uniform reference weights *w*_0,*i*_ = const. for the unbiased ensembles produced by HCG. The reference weights of the individual chains were then minimally adjusted such that the ensemble average better agrees with the experimental observable, while making sure that the refined ensemble was well-defined and converged. The confidence parameter θ in BioEn^37,38^ was chosen by L-curve analysis. As a measure of the extent of reweighting, we used the Kullback–Leibler divergence *S*_KL_ = *∑*_*i*_*w*_*i*_ ln(*w*_*i*_/*w*_0,*i*_) between the reference weights *w*_0,*i*_ and the refined weights *w*_*i*_, both normalized, *∑*_*i*_*w*_0,*i*_ = *∑*_*i*_*w*_*i*_ = 1. In addition, we inspected the cumulative distribution function (CDF) of rank-ordered weights *w*_*i*_. A rapid initial rise indicates that few ensemble members carry a large fraction of the weight, which in turn indicates poor overlap between reference and refined ensembles.

### 2.6 Analysis of ssRNA Conformations

The python packages Barnaba,^55^ MDTraj,^56^ and MDAnalysis^57,58^ were used to perform analyses of the ssRNA conformations.

A cluster analysis was performed using the Barnaba software^55^ exemplary of the rA_4_ fragment conformations as sampled in 100 ns MD simulation trajectory. The sampled conformations within 10000 frames were assigned to 6 different clusters. In the cluster analysis, we first calculated the g-vectors describing the relative positions of the nucleotide pairs in each structure. We then performed a principal component analysis (PCA) of the g-vectors, projecting the data points onto the plane of the first and second principal component axes. The clustering was performed via a Barnaba wrapper of the DBSCAN function from the scikit-learn package. Here, we set the minimum distance for nearest neighbors to eps = 0.35 and the minimum number of samples per cluster to 50.

#### Quantification of Base Stacking

We used the Barnaba software^55^ to screen the assembled structures for stacked bases. For each base, we quantified the stacking by a factor *q*_stacking_. For nucleobases not involved in any stack, we set *q*_stacking_ = 0. For stacks of *n*_stacked_ = 2 and 3, we set *q*_stacking_ = 0.25*c* and *q*_stacking_ = 0.5*c*, respectively, where *c* = *n*/(*n* – 1) with *n* the total number of nucleobases in the ssRNA. For longer consecutive stacks with *n*_stacked_ ≥ 4 bases, we set *q*_stacking_ = *c* – *c*/(*n*_stacked_ – 1). We then used *q*_stacking_ to color the bases in the structural visualizations (Figure 2).

### 2.7 Calculation of Experimental Observables

#### FRET

We calculated FRET efficiencies for the rA_19_ ssRNA ensembles obtained by HCG with explicit dyes attached at the 5′ and 3′ ends. The interdye distance *r* was calculated as the geometric distance between the central oxygen atoms of the two FRET dye labels,^21^ as determined using MDAnalysis.^57,58^ For the orientational factor κ^2^ in the Förster theory, we considered three models that differed in their assumptions on the dye dynamics. A similar approach has been employed before.^59^

In model 1, we set κ^2^ = 2/3,^40,60^ assuming implicitly that dye rotation is isotropic and fast^61,62^ compared to the fluorescence lifetime of the donor, which in the absence of the acceptor is τ_*D*_ ≈ 4 ns.^21^ The transfer efficiency *E* of an individual ssRNA conformation labeled with fluorophores at each end was then calculated as
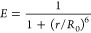
1The Förster radius *R*_0_ was set to the experimentally determined value of *R*_0_ = 5.4 nm.^21^ In model 2, we assumed also the dye linker dynamics to be fast and accordingly attached ≈20 conformers for the dye pairs to a given ssRNA conformation, averaged the interdye distance *r* over these conformers, and then calculated the FRET intensity according to eq 1 with the average *r*. By contrast, in model 3, we assumed the dye dynamics to be slow. Accordingly, we determined both *r* and κ^2^ explicitly for each dye-labeled ensemble member. We calculated κ^2^ as
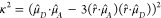
2where  and  are unit vectors in the direction of the transition dipole moments of donor and acceptor, respectively, and *r̂* is a unit vector pointing in the direction between the central oxygen atoms of the two dyes. We then calculated the rate of energy transfer as  and the FRET efficiency of each ssRNA conformation in the ensemble as^63^
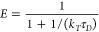
3For all three models, the efficiency *E* was then averaged over the ssRNA conformations in the ensemble with their respective weights. The three models can be considered as extremes with respect to the assumed dye dynamics. Importantly, in all models, we assumed the ssRNA dynamics to be slow compared to the fluorescence lifetime τ_*D*_.

For model 2, we mapped FRET labels onto the full-length rA_19_ grown for analysis with model 1, with labels integrated in the models at the fragment level. Particularly, we randomly picked an rA_19_ conformation *i* and attempted to simultaneously replace both labels with randomly picked label conformations of Alexa Fluor 594 *j* and Alexa Fluor 488 *k*. Here, we followed the procedure for the heavy atom alignment and clash search as described above for the dye mapping on the fragments. In case of a steric clash or if the RMSD exceeded 0.8 Å in the alignment, both dye conformations were discarded, and a new pair of dyes was drawn. We attempted to replace the dye conformations 1000 times for each of the 10000 randomly drawn rA_19_ conformations. The normalized acceptance rate for dye replacements determined for conformation *i* was then used as the weight for ⟨*r*_*i*_⟩ for each conformation in the ensemble.

#### SAXS

For each ensemble member *i* of either the rA_30_, rU_30_, or rUCAAUC HCG ensembles, we calculated the SAXS scattering intensity *I*_*i*_(*q*) at scattering vector *q* using Crysol^64^ following ref (14). The calculated scattering intensities *I*_*i*_(*q*) with normalized weights *w*_*i*_ (i.e., *∑*_*i*_*w*_*i*_ = 1) were averaged over the ensemble as *I*_sim_(*q*) = *∑*_*i*_*w*_*i*_*I*_*i*_(*q*). In the limit of *q* → 0, the Guinier approximation becomes exact, *I*_*i*_(*q*) ≈ *I*_0,*i*_ exp(−*q*^2^*R*_*G*,*i*_^2^/3), where *I*_0,*i*_ is the intensity at *q* = 0, and *R*_*G*,*i*_ is the radius of gyration of ensemble member *i*. Accordingly, we calculated *R*_*G*,*i*_ from the slope of ln *I*_*i*_(*q*) with respect to *q*^2^ at *q* = 0 as
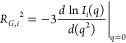
4We evaluated the slope as a numerical first difference. With *I*_*i*_(*q*) ≈ *I*_0,*i*_ exp(−*q*^2^*R*_*G*,*i*_^2^/3) at small *q*, *I*_sim_(*q*) = *∑*_*i*_*w*_*i*_*I*_*i*_(*q*), and *I*_0,*i*_ being nearly constant, we determined the root-mean-square (RMS) *R*_*G*_ by averaging over the ensemble
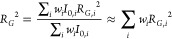
5We related the calculated intensity *I*_sim_(*q*) to the measured intensity *I*(*q*) by performing a least-squares fit of *I*(*q*) = *aI*_sim_(*q*) + *b* with an intensity scaling factor *a* and a constant background correction factor *b* as fit parameters. To assess the quality of the fit, we calculated the reduced chi-squared normalized by the number *M* of data points
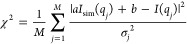
6with σ_*j*_ the reported experimental standard error of *I*(*q*_*j*_). Values of χ^2^ ≲ 1 indicate agreement within the experimental uncertainty. We used an implementation of BioEn specific for SAXS data that fits nuisance parameters globally for the ensemble average during the refinement, with the code available at https://github.com/bio-phys/SAXS_BioEn/. In brief, we performed an initial fit of the intensity scale factor *a* and background correction *b*, which were then updated using the refined weights until convergence was achieved.

We assessed the quality of the ensembles with the χ^2^ statistic for the squared residuals and the hplusminus statistic for the sign-order of the residuals, calculating *p*-values for both tests individually and in combination.^65^

## 3 Results and Discussion

### HCG Produces Broad Structural Ensembles of ssRNA

We used HCG to sample structural ensembles of ssRNA polymers with four different sequences: rA_30_, rA_19_, rU_30_, and rUCAAUC. For all four systems, we observed a combination of extended and compact conformations, as shown representatively in Figures 2 and S3. Compactness is associated with kinks in the ssRNA backbone (see, e.g., bottom center rA_30_ structure in Figure 2). In the more extended structures, the chains retained features of the A-form helix (e.g., rA_30_ top left in Figure 2). In particular, we observed stretches of continuously stacked nucleobases.

The ssRNA structure in the HCG ensembles depends on the nucleotide sequence. Whereas the poly purine rA_30_ tends to form relatively straight segments of stacked adenines, poly pyrimidine rU_30_ is visually rather distorted with stretches of unstacked uridines (Figure 2). A stacking analysis using Barnaba showed about four fewer stacks in rU_30_ than rA_30_ on average (Figure S4A). Here, a single stack was defined as two nucleobases with particular distances and orientation to each other.^55^ We further looked at consecutively stacked nucleobases, which we defined as four or more stacked nucleobases in a row and colored the nucleobases accordingly (Figures 2 and S4B). Key to retaining base stacking in the fragment assembly was the inclusion of the atoms of the nucleobase in the RMSD alignment, which ensured that the relative base–base orientation of the fragments was retained in the HCG assembly (Figure 1). The observed sequence dependence is in line with the behavior previously reported for the ssRNA polymers investigated here.^14,16,18,21,66^

The structure in long ssRNA chains is reflected in the fragment libraries used for HCG. We clustered the rA_4_ fragment library according to their structure. In the largest clusters, stacked A-form like conformations dominate, either with perfectly stacked nucleobases (cluster 0 with 42%) or with nucleotide 4 inverted (cluster 1 with 39%; see Supplementary Figure S1). NMR studies support the presence of a substantial fraction of A-form like conformations for short single stranded RNA fragments.^9,10,67^ The next largest clusters 2 and 3 are sparsely populated (≈1%), containing structures with A3 unstacked and A4 inverted (cluster 2), and all bases being unstacked (cluster 3).

HCG assembly also largely preserves the distribution of torsion angles in the fragment libraries, as shown for rA_19_ in Figure S5. In the HCG assembly, the central two nucleotides at positions 2 and 3 of the tetrameric fragments were retained. Figure S5 compares the distributions of the backbone torsions averaged over the 19 bases in each A2 and A3 as sampled in rA_19_ chains to the respective distributions in the fragments. First, we found that the distributions are essentially independent of the position in the HCG assembly, as would be expected for a long homopolymer. Second, we found that HCG largely retained the torsion angle distributions of the fragments. However, we observed that some populations were altered or completely vanished. The small differences reflect in part actual incompatibilities with longer chains yet also choices in HCG, in particular of the atoms to align and of the clash criteria. Differences as, e.g., in α for A3 or ϵ and ζ for both A2 and A3 may be amplified by the different nature of the preceding nucleotide at the 5′ position and the following nucleotide at the 3′ position of A2 or A3, respectively. In particular, in the MD fragments these nucleotides were attached to terminal nucleotides, which may impact the distribution of torsional angles at the P - O5′ bond (α), C3′ - O3′ bond (ϵ), and the O3′ - P(+1) bond (ζ).

The recovery of the torsional distributions after assembly suggests that the local and global structural features observed in the assembled full-length chain arose from the local structure sampled in the fragments, as found before for tau K18.^40^ In particular, rA_4_ fragments sampled in the DESRES force fields mostly exhibited an A-form helix-like conformation with a considerable population of conformations with one or two of the 4 nucleotides being unstacked (see Figure S1 clusters 1 and 3). In turn, this resulted in either pseudo A-form helix-like populations as well as populations of kinked or looplike structures for ssRNA polymers sampled with HCG, with some of the chains even featuring patterns with bulging nucleobases (Figure 2 and Figure S3).

### ssRNA from HCG Reproduces SAXS Data

**Figure 3:**
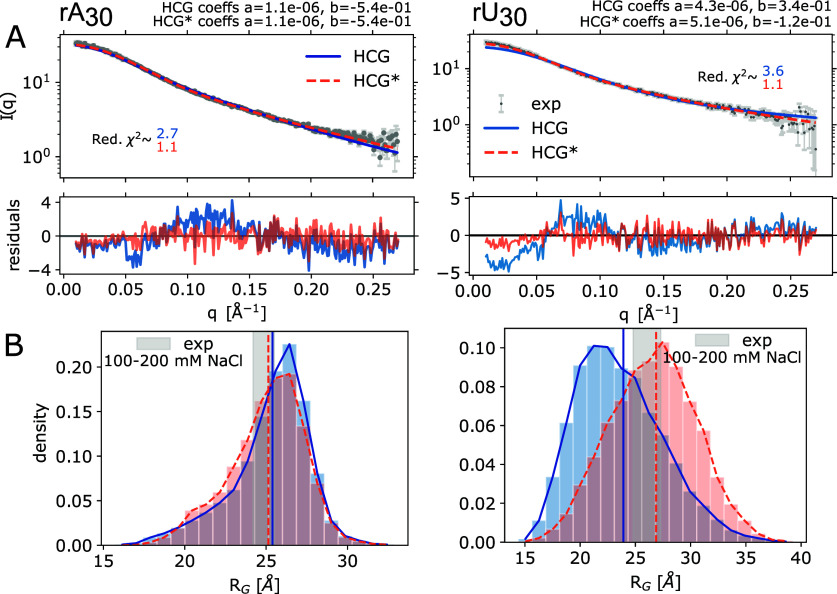
Comparison of the rA_30_ and rU_30_ HCG structural ensemble to SAXS measurements^18^ (left and right columns, respectively). (A) Top: Experimental SAXS profiles measured at 100 mM NaCl in gray and the average profile calculated using Crysol^64^ for the unrefined HCG ensembles (blue), 10000 structures each, and the refined HCG* ensembles (orange) with weights for θ = 100 and θ = 46 for rA_30_ and rU_30_, respectively. Intensity scale factors *a* and background correction constants *b* determined by least-squares fitting are shown in the plots. Bottom: Residuals. (B) Distribution of *R*_*G*_ in the unrefined HCG and reweighted HCG* ensembles (blue and orange, respectively). Vertical lines indicate the RMS *R*_*G*_ value of the HCG ensemble (solid blue) and the weighted RMS *R*_*G*_ value (HCG*, dotted orange). The gray shaded area highlights the area spanned by the *R*_*G*_ value inferred from the SAXS profiles measured at 100 mM and 200 mM NaCl including the error range.

We compared the calculated SAXS intensity profiles obtained by averaging across the rA_30_ and rU_30_ HCG ensembles to experimental profiles measured at 100 mM NaCl^18^ (Figure 3A). The rA_30_ and rU_30_ ensembles were assembled from rA_4_ and rGUUU fragment libraries, respectively (see Methods). Overall, the agreement was good, with reduced χ^2^ errors (mean-squared residuals divided by the experimental error) of 2.7 and 3.6, respectively. However, the residuals revealed small but systematic deviations for both polymers. In the relatively featureless intensity profiles, the residuals pointed to somewhat too extended structures for rA_30_ and too compact structures for rU_30_.

Considering the fact that HCG does not account for long-range electrostatic interactions and salt screening effects beyond the scale of the fragments, the agreement between measured and calculated SAXS intensities at ∼100 mM NaCl is remarkably good. Solvent and, in particular, ions affect the global structure of the negatively charged nucleic acids polymers.^9,11,16,17,21,24,68^ At the high concentrations of the SAXS experiments, interchain interactions may also be relevant.^18,69^

### Gentle Ensemble Reweighting Further Improves Agreement with SAXS Data

We refined the HCG ensembles of rA_30_ and rU_30_ by performing BioEn reweighting^37,38^ against the experimental scattering profile measured at 100 mM. Using a rather gentle bias, we adjusted the weights of the ensemble members to agree with the experimental profile with reduced χ^2^ values of ≈1.1 for both polymers (HCG* ensemble in Figures 3A, S6 light orange, and S7).

We also found good agreement between the measured and calculated values of the radius of gyration, *R*_*G*_. We calculated the RMS *R*_*G*_ = ⟨*R*_*G*,*i*_^2^⟩^1/2^ as an average over members *i* of the ensemble with their respective weights. For rA_30_, the RMS average *R*_*G*_ over the HCG ensemble was within the uncertainty of the *R*_*G*_ measured by SAXS at 100 and 200 mM NaCl;^18^ for rU_30_, it was just below the expected range (Figure 3B). In this range, salt concentration was found to have only a small effect on *R*_*G*_.^18,69^ Reweighting by BioEn to match SAXS intensities *I*(*q*) also improved the agreement of the calculated and measured *R*_*G*_ values. Overall, HCG captured the global dimensions of rA_30_ and rU_30_, and a gentle BioEn reweighting resulted in near-perfect agreement at higher NaCl concentrations for rA_30_ (see below).

### Dependence on Fragment Library

For homopolymeric ssRNA, using a homopolymeric fragment (here: rA_4_) is a natural choice that facilitates the combination of fragments into longer chains, as the base overhangs at the 5′ and 3′ ends of the fragments to be combined are then identical. By contrast, to assemble generic ssRNA sequences by HCG, it is advantageous to fix the base at the 5′ end to minimize the number of required fragment sequences. For the tetramer fragments used here, with sequence rGXYZ, we then have only 4^3^ = 64 sequences to consider. Here, having built both rA_4_ and rGA_3_ libraries, we can directly compare the two approaches to identify possible issues in HCG. We found that the distributions of *R*_*G*_ for rA_30_ chains grown by HCG with rA_4_ fragments and with rGA_3_ fragments show negligible differences (Kullback–Leibler divergence *S*_KL_(rGA_3_∥rA_4_) = 0.01; see Figure S8), indicating that both libraries produce very similar results.

### rA_30_ HCG Ensemble Reproduces SAXS Profiles Measured at Different Salt Concentrations after Gentle BioEn Refinement

In their study on the salt dependence of ssRNA rA_30_ and rU_30_, Plumridge et al.^18^ have shown that the global structure of these highly charged polymers depends on (i) the concentration of ions in the solvent and (ii) the ion type. Here, we compared the rA_30_ HCG ensemble, grown from rA_4_ fragments simulated at 150 mM NaCl, to their SAXS profiles measured at 20, 100, 200, 400, and 600 mM NaCl concentrations. Despite the fact that we grew the polymer via HCG without taking into account long-range electrostatics beyond the fragment level, the HCG profile matched experimental SAXS profiles recorded at different salt concentrations reasonably well even without reweighting (Figures S9–S13A blue). For reference, we least-squares fitted the unrefined SAXS profile of the rU_30_ produced by HCG to the salt dependent experimental SAXS profiles of rA_30_, adjusting only the intensity scale factors *a* and the background corrections *b*. For all salt concentrations, the HCG profiles for rA_30_ gave a better fit to the rA_30_ experiments than the HCG profiles for rU_30_, albeit with only small differences in χ^2^ (Figure S14 and Supporting Table S1). Plumridge et al.^18^ reported on the rather small differences in the global shape found for both polymers for salt concentrations > 20 mM. For 100 mM NaCl, for instance, we found the experimental profiles of rU_30_ and rA_30_ to agree with reduced χ^2^ ≈ 0.91 after fitting *a* and *b* (Supporting Table S1).

**Figure 4:**
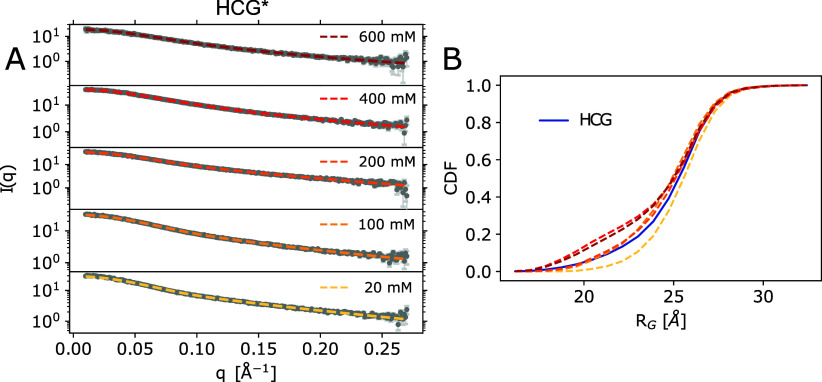
SAXS measurements of rA_30_ at different salt concentrations. (A) SAXS profiles from the rA_30_ HCG ensemble refined against experimental profiles measured at 20, 100, 200, 400, and 600 mM NaCl. HCG* is shown in yellow to dark red. Experimental profiles are shown in gray; errors are shown in light gray. (B) Cumulative distribution of *R*_*G*_ as predicted for HCG using Crysol^64^ in blue and the weighted distributions using the refined weights.

BioEn reweighting of the HCG ensemble against the scattering profiles measured at the respective salt concentration established nearly perfect agreement with the experimental profiles (Figure 4A, Figures S9–S13A orange). For each salt concentration, we chose a set of weights for the regularization parameter θ = 100 resulting in reduced χ^2^ < 2. According to the L-curve analysis, with Kullback–Leibler divergences close to zero and the cumulative distribution functions (CDF) of rank-ordered weights staying close to uniform reference weights, all BioEn reweightings placed a rather gentle bias on the initial ensemble (Figure S6).

The reweighted RMS *R*_*G*_ values for the rA_30_ ensemble were shifted toward the experimental values for each concentration (Figures S9–S13C). The shape of the *R*_*G*_ distributions was minimally modified when we applied the refined weights for 20, 100, and 200 mM (Figures 4B left column and S9, S10, and S11C). In fact, for 100 and 200 mM NaCl, the RNA conformations of rA_30_ with *R*_*G*_ < 20 Å lost weight against conformations with 20 Å < *R*_*G*_ < 25 Å (Figures 3B left column and S11C). For the highest salt concentrations of 400 and 600 mM NaCl, a distinct shoulder developed in the reweighted *R*_*G*_ distribution at *R*_*G*_ ≈ 20 Å (Figures S12 and S13C). The diminished role of electrostatic repulsion between ssRNA phosphate groups at high salt concentration may explain this trend to compaction.

We further assessed the quality of the *I*(*q*) fits using the hplusminus test statistic for ordered data.^65^ Applied to the scattering intensities *I*(*q*), it tends to pick up indications for systematic errors, e.g., as a result of deviations in the global size and shape of the ensemble members. Here, going in the HCG* ensembles from low to high salt concentration, we found that systematic deviations at small *q* values decreased, as judged by the residuals and a screening of their signs. In return, we found improved *p*-values for the reweighted ensembles at higher salt concentrations (Figures S9–S13A and B).

Despite the overall efficient reweighting of the HCG ensemble resulting in almost perfect agreement with experiment, we emphasize that the refined ensemble lacks information about electrostatics and other interaction. For a more detailed assessment, one could perform additional MD simulations at the respective salt condition using a small subset of the models sampled in the HCG ensemble as start structure and choosing a reasonable force-field.^28,43^ Such simulations would provide information about electrostatic interactions and the solvent layer.

### HCG Ensembles of rUCAAUC Capture SAXS and NMR Experiments

**Figure 5:**
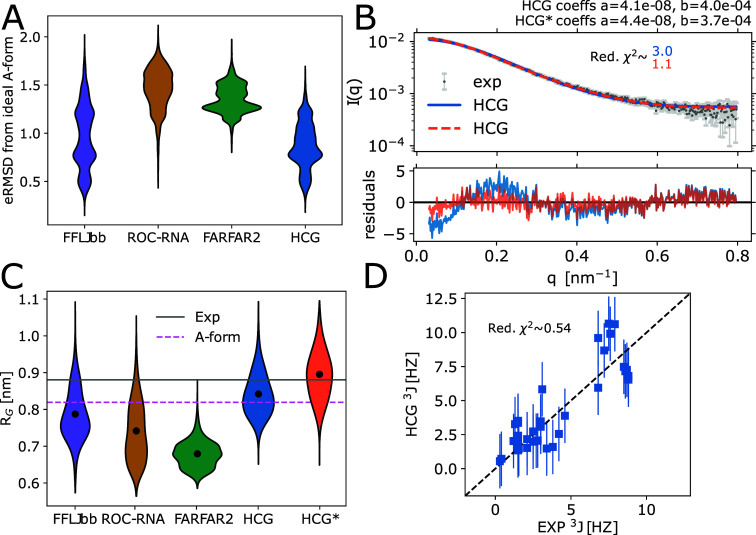
Characterization of structural ensembles of heteropolymeric rUCAAUC sampled with MD and FARFAR2 from ref (14) and HCG. (A) Distribution of the eRMSD to ideal A-form. (B) Top: Average SAXS profile calculated for the HCG ensemble before (blue) and after ensemble refinement (HCG* with weights for θ = 100 in orange) fitted to the experimental profile^14^ (orange and gray, respectively). The intensity scale factors *a* and the background correction constants *b* as calculated by least-squares fitting are shown in the plot. Bottom: Residuals calculated for scattering profiles. (C) Distribution of *R*_*G*_ values in sampled ensembles, the refined HCG* ensemble, experimental average as determined from SAXS, and for typical A-form. (D) Correlation plot of experimentally measured ^3^*J* couplings of the backbone and sugar moiety^66^ and calculated values for HCG. Vertical bars indicate the estimated uncertainty of ±2 Hz (one standard error) in calculating the ^3^*J*-couplings from RNA structures using approximate Karplus relations.^13,66^

Using the heterotetramer fragment library for HCG, we are able to grow heteropolymeric ssRNAs of an arbitrary sequence. In the following, we show results for the rUCAAUC hexamer, which has been investigated previously by experiments and MD simulations.^14,66^ Visually, the structures appeared rather extended, albeit with populations of structures in which one or two nucleotides were unstacked, similar to what was observed by Bergonzo et al.^14^ (Figures 2 and 5A).

Judging from the comparison to the published SAXS data,^14^ the HCG ensemble of rUCAAUC chains captured the global dimensions without any refinement. The average SAXS profile calculated for the HCG ensemble was in good agreement with the experimental profile, with a reduced χ^2^ of about 3.0 and small deviations at small *q* (Figure 5B). For reference, Bergonzo et al.^14^ found profiles of similar quality in their MD simulations of full-length rUCAAUC. For the LJbb force field, their agreement with the SAXS experiments was slightly better, with χ^2^ ≈ 2.4 and the deviations at small *q* being less pronounced.

The distribution of *R*_*G*_ in the HCG ensemble was in line with the distribution in the MD ensemble sampled with the LJbb force field (FFLJbb) by Bergonzo et al.^14^ (Figure 5C). The RMS *R*_*G*_ as calculated for the HCG ensemble was slightly closer to the experimentally determined value than that of the ensemble from full MD simulations. Interestingly, the RMS *R*_*G*_ is close to that of an rUCAAUC polymer in an ideal A-form helix conformation. Overall, the conformations sampled with HCG seemed to resemble a typical A-form to a larger extent than conformations sampled with the other approaches shown here, with the eRMSD from a typical A-form being smaller on average (Figure 5A).

The analysis of NMR ^3^*J* couplings of the backbone and sugar moiety revealed that overall HCG sampled local properties, as reflected in the torsion angles of the sugar moiety as well as the nucleic acid backbone, in excellent agreement with the experiments (Figure 5D). Small deviations in the calculated ^3^*J* couplings were within their predicted uncertainty (≈2 Hz for the Karplus relation used to calculate the scalar coupling^55^), resulting in a reduced χ^2^ value of ≈0.54. Thus, we found our ensemble to agree with experiment as good as the MD ensemble (LJbb force field) from Bergonzo et al.^14^ We note that for the experimental values, Zhao et al. suggested an error of 2 Hz as well due to the deviations of measured values in multiple independent measurements (see ref (66) and Table S4), which we did not consider here.

We reweighted the SAXS profiles calculated for HCG against the experimentally measured scattering profile. Using a small bias with weights for θ = 100, we found almost perfect agreement with the experimental profile with reduced χ^2^ ≈ 1.1 and *S*_KL_ ≪ 1 (Figure S15A, B). Deviations we observed for the refined profile were within the experimental error range, and only very small deviations for *q* < 0.1 nm. The refined weights were used to calculate weighted distributions and averages for properties we analyzed here. The weighted distribution of *R*_*G*_ values was shifted toward larger values with the weighted RMS *R*_*G*_ value being in perfect agreement with the experimental value. Interestingly, we observed only small deviations from ideal A-form and scalar couplings close to experiment (Figure S15C, D).

### HCG Produces Ensembles with a Large Conformational Variability

We observed a high diversity of global dimensions (e.g., for rU_30_ in Figure 3, right panel). Using larger fragment sizes to prepare a fragment library, e.g., pentamers with the central trimers and the 3′ terminal capping nucleotide being flexible and the 5′ terminal cap fixed, would still be computationally feasible, with 4^4^ = 256 fragments. It is interesting to speculate if we may be able to sample a higher population of structures that feature important local motifs, by sampling more local interactions within the input MD fragments.

We compared the sampled structural diversity in HCG and MD ensembles in terms of pairwise RMSDs, calculated by using all heavy atoms within a polymer. For short chains (tetramers and hexamers), the distribution of the pairwise RMSD within MD ensembles was slightly larger than within the HCG ensemble. This suggests that a slightly larger conformational variability was sampled with MD (Figure S16), at least in terms of the pairwise RMSD. The pairwise RMSDs between MD and HCG were distributed around 4 nm (Figure S16, rose), similar to what we observed for two independent HCG ensembles of the same polymer (Figure S16C, dashed gray). For rA_4_, all distributions were shifted toward smaller pairwise RMSDs, probably due to the large population of A-form like helix conformations (Figures S1 and S16A).

Importantly, we do not know the actual extent of structural variability or the expected distribution of pairwise RMSDs for a native ssRNA ensemble. For ensemble refinement, however, it is advantageous to have a broad sampling that covers the relevant conformation space. By integrating experimental information, ensemble refinement methods such as BioEn^37,38^ then down-weight conformations with low statistical relevance. By contrast, if the starting ensemble does not cover the relevant conformation space, conformations in this region would have to be added by biased sampling for a proper ensemble refinement.

In general, efficient comparisons of structurally heterogeneous ensembles are difficult.^27^ Several algorithms exist to cluster ensemble members according to different properties, often accompanied by machine learning techniques. However, finding appropriate collective variables that really capture the important properties needed to display the differences between ensembles is not straightforward. Recently, a tool to compare structural ensembles of IDPs by determining differences in distributions of local and global properties of the conformations based on a Wasserstein metric was introduced.^70^

### ssRNA Polymers Grown with HCG Can Be Combined with dsRNA

**Figure 6:**
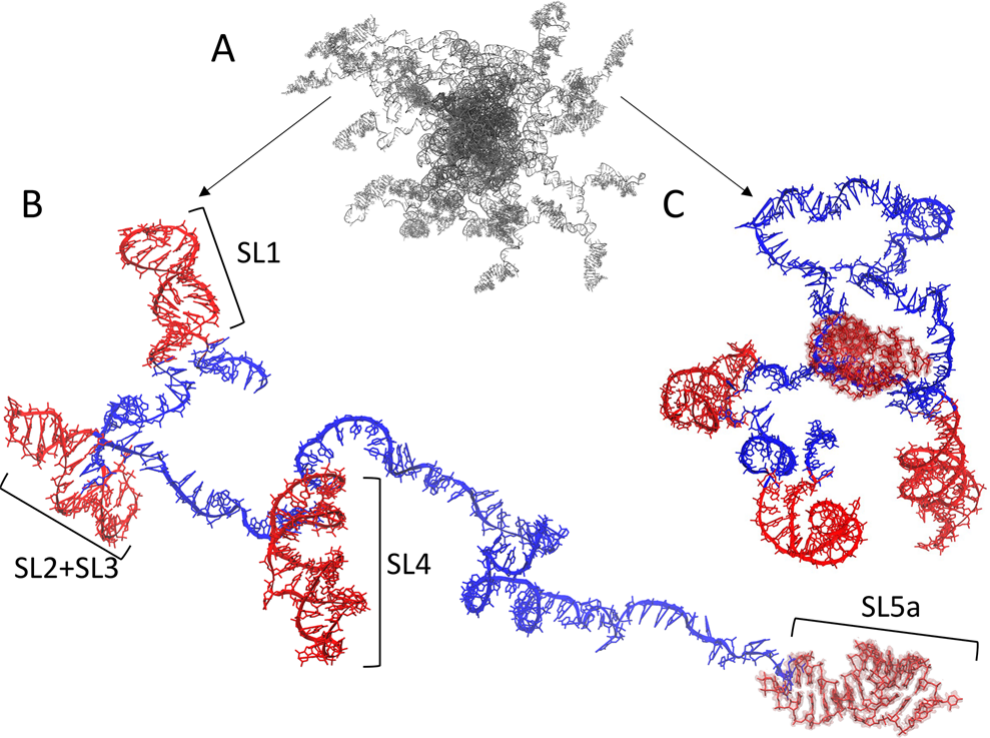
Model of the 5′ UTR region of SARS-CoV-2 genomic RNA built by HCG. (A) Ensemble overview. (B) and (C) show two representatives of extended and compact conformations, respectively, with more detail. Structures of the five stem-loop regions drawn at random from MD trajectories^53^ are shown in red. The connecting single-stranded RNA is shown in blue. The structures are shown with atomic detail. Hydrogen atoms are omitted for clarity. The backbone atoms are shown in a cartoon representation except for the last stem-loop at the 3′ end, which is highlighted as a surface. The full-length structure shown here covers 233 nucleotides.

Conformations sampled with HCG can be easily combined with structured dsRNA or other ordered structures. Here, we exemplarily modeled a region of the 5′ UTR of the SARS-CoV-2 genomic RNA (sequence shown in Figure S2). Structured stem-loops were taken from earlier studies using FARFAR2^53,71^ and from NMR studies.^54^ To model the 5′ UTR, we used MD trajectories of five stem-loops provided by Bottaro et. al^53^ as input ensembles for the structured parts (see Methods). The stem-loop, highlighted in red, was then connected by single-stranded regions grown with HCG, highlighted in blue, resulting in a model containing 233 nucleotides in total. The final ensemble with about 44 different conformations features models with more extended and more compact single-stranded regions, dictating the overall global dimensions of the modeled 5′ UTR (see Figure 6A). For illustration, we randomly chose two structures (Figure 6B and C, respectively).

We demonstrated here a possible application of HCG to model structures of RNA molecules that combined structured and unstructured regions, such as mRNA molecules. More generally, a similar scheme may be applied to model any kind of biomolecule featuring unstructured parts, e.g., by adding a polyA tail to mRNA. Importantly, since HCG is modular, we can either add additional assembly steps and assemble the different regions after the initial growth or grow the flexible chain with HCG directly at the structured biomolecule. In particular, such models can be used for further analysis, e.g., as an initial structure for MD simulations.

### HCG Ensembles of rA_19_ with Mapped Dyes Are Somewhat too Extended on Average as Judged by Experimental FRET Efficiencies

We calculated FRET efficiencies for the HCG ensemble of rA_19_ and compared them to the measured mean FRET efficiency ⟨*E*⟩ = 0.56 ± 0.03 obtained in single-molecule FRET experiments at 150 mM NaCl concentration.^21^ For model 1 with fast and isotropic averaging for the dye orientations about fixed dye positions (eq 1), we obtained a mean efficiency of ⟨*E*⟩ ≈ 0.41. In model 2, we start from the ensemble in model 1 but with multiple dye pair conformations placed onto every conformation *i* in the rA_19_ ensemble. By averaging the FRET efficiency over these dye pairs and their orientations, we effectively assumed dynamic dyes in model 2, which we consider to be more realistic than model 1. We observed a mean FRET efficiency of ⟨*E*⟩ ≈ 0.42. In the less realistic model 3 with fully static dyes, we fixed interdye distances and determined κ^2^ explicitly from the dye conformations (eq 3). For model 3, we obtained ⟨*E*⟩ ≈ 0.32 with a high population of conformations having *E* < 0.1. By comparison, MD simulations of full-length rA_19_ using the same force field as in our fragment MD simulation gave FRET efficiencies of ⟨*E*⟩ ≈ 0.3,^21^ calculated with explicit dyes mapped onto the sampled conformers and κ^2^ = 2/3 fixed, as in our model 1. Differences we observed for FRET efficiencies calculated from the MD and the HCG ensemble using model 1 may indicate that the MD simulation was too short, with 7 μs of sampling in aggregate. Alternatively, we may have a favorable compensation of errors in chain growth by accounting primarily for the local structure.

**Figure 7:**
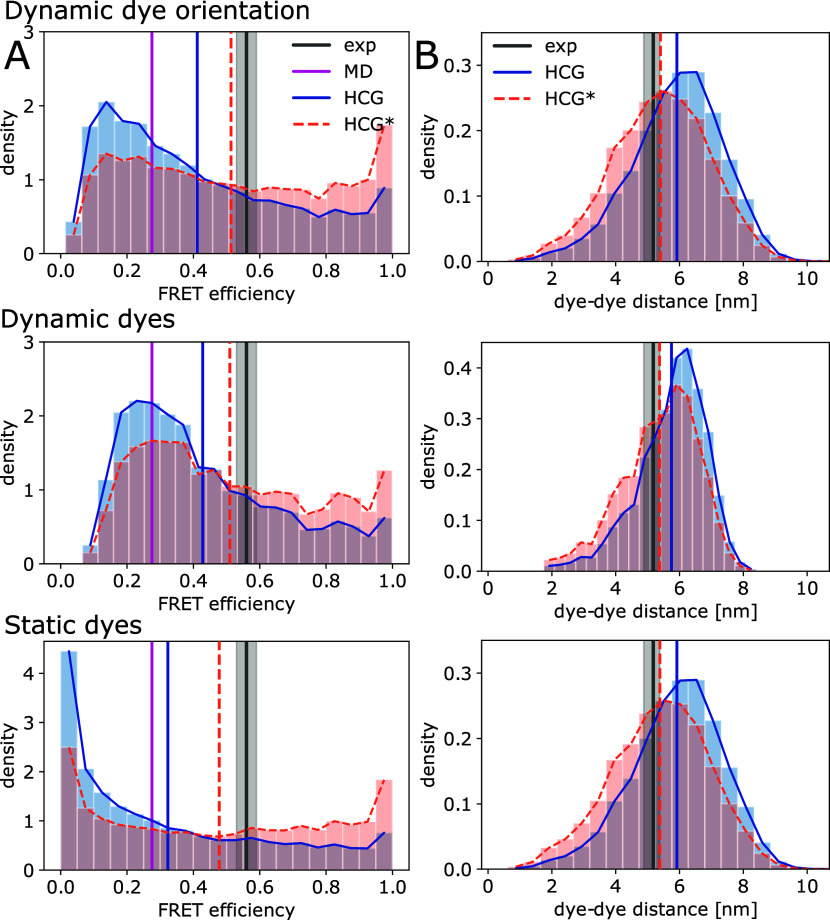
HCG ensembles of rA_19_ compared to single-molecule FRET experiments. (A) Distribution of FRET efficiencies in the HCG ensemble (blue) and in the reweighted HCG* ensemble (orange) calculated with model 1 (top) with dynamic dye orientations and κ^2^ = 2/3, model 2 (middle) with dynamic dyes and κ^2^ = 2/3, and model 3 (bottom) with static dyes. For HCG*, we chose refined weights for θ = 40 (models 1 and 2) and θ = 60 (model 3). Vertical lines indicate the mean FRET efficiency measured in experiment (black, with gray shading indicating ± SEM), sampled in MD simulations of full-length rA_19_ with the same force field as used here to build fragment libraries^21^ (magenta), and calculated for the HCG (blue) and HCG* ensembles (orange). (B) Distributions of the interdye distance as determined for the HCG ensemble (blue) and the reweighted HCG* ensemble (orange) with models 1 (top), 2 (middle), and 3 (bottom). The mean distances for experiment (black), MD simulation (magenta), HCG (blue), and HCG* (orange) are shown as vertical solid and dotted lines. The experimental mean interdye distance was inferred from experimental single-molecule FRET efficiencies using a worm-like-chain model for the distance probability density function.^21^

Using BioEn,^37,38^ we then gently reweighted the ensembles of models 1, 2, and 3 to match the experimental mean FRET efficiency. For model 1 and model 2, we obtained reduced χ^2^ ≈ 1.4 for a BioEn confidence parameter θ = 40, and for model 3, we obtained χ^2^ ≈ 2.3 for θ = 60 (see Figure 7A orange top row, middle row, and bottom row, respectively). The ensemble refinement assigned higher weights to the tail of the ensemble, i.e., to more compact chains with *E* > 0.6. In turn, the weighted distributions and the average interdye distance were shifted to shorter distances within the range inferred from the single-molecule FRET experiment using a worm-like chain polymer model (see Figure 7B). Here, either the structures were more compact, the mapped dyes featured less extended linkers, and/or the mapped dyes pointed toward each other (Figure S3). This shift in population toward more compact structures is qualitatively consistent with what we found in the BioEn reweighting for rA_30_ according to the SAXS data (Figure 3B). However, the shift there was considerably smaller, as the *R*_*G*_ value had already agreed with the measurements within the uncertainty. We note that the *r* distributions in the HCG* ensembles for models 1 and 3 are nearly identical (Figure 7B). For model 2, the distance distribution in HCG was narrower, and for HCG*, the peak of the distribution was slightly shifted toward larger interdye distances. A small shoulder at around 4 nm dye–dye distance, present in HCG* for all models, was more pronounced.

The shape of the reweighted distributions of the FRET efficiency may indicate slight overfitting. However, judging from the L-curve analysis and the CDF of rank-ordered weights (Figure S17, orange, dark green, and dark red), the set of weights we chose seemed to impose a rather gentle bias with *S*_KL_ < 0.2 for all three models. An important point to consider is how to properly perform the ensemble reweighting for polymers with attached labels.^39,72^ In approaches such as FRETpredict,^73^ dyes are placed onto proteins using a rotamer library approach (RLA) to predict FRET efficiencies with individual statistical weights. In the present study, we reweighted the whole molecule, i.e., polymer chain plus the attached fluorophore molecules. As an alternative, one could reweight the chain and dye separately.

### HCG Compared to MD Simulations of rU_40_ Using the Anton Supercomputer

We compared the distributions of the radius of gyration *R*_*G*_ and the O5′-O3′ distance in an HCG ensemble of rU_40_ to those sampled in ∼100 μs MD simulation runs of full-length rU_40_ with the DESRES (DES-Amber0.9) force field^23^ at 0.05, 0.1, 0.5, and 1.0 M NaCl and a newer, modified version DES-Amber3.2^74^ at 0.05, 0.1, 0.2, 0.4, and 0.5 M NaCl. For both force fields, rU_40_ transitioned between an extended state and a more compact state with folded-back conformations.^23,74^ The population of the compact state increased with increasing salt concentration. For the rU_40_ polymers assembled by HCG from MD fragments sampled at 0.15 M NaCl with the DESRES force field, the distributions of *R*_*G*_ and the O5′-O3′ distance are intermediate between the distributions in full MD simulations with the DES-Amber0.9^23^ and DES-Amber3.2^74^ at the closest NaCl concentrations below (0.1 M) and above (1.0 M, 0.2, and 0.4 M, respectively; see Figure S18). Overall, the HCG ensemble covers a range of *R*_*G*_ values similar to that of the MD simulations. Together with our results for rU_30_ compared to the experimental SAXS data^18^ (see Figure 3, right column), we conclude that HCG performs well for polymeric rU_*n*_ chains.

The HCG ensemble for rU_40_ also compares well to the experiments without adjustments. Chen et al.^16^ have determined mean end-to-end distances of ≈66 Å and ≈64 Å for 100 and 200 mM NaCl, respectively, from FRET measurements. The mean end-to-end distance in the HCG ensemble of rU_40_ at 150 mM NaCl is ≈67 Å, very close to the experimental values.

The consistency of HCG and full MD simulations for rU_40_ with state-of-the-art force fields is reassuring because there are important differences between the two, even if they are conducted with the same force field. Whereas HCG uses RNA fragment libraries built by MD simulations, only steric interactions are considered between distant fragments so that ssRNA structures folding back onto themselves have no stabilizing interactions. Structures such as the stem-loops in the SARS-CoV-2 5′ UTR (Figure 6) can be included in HCG as fragments, as shown here. However, folded-back structures of ssRNA can also be an artifact of the MD simulation force field, which was a major driver for the development of HCG fragment-based methods, e.g., for the modeling of fluorophore labeled ssRNA.^21^ In terms of sampling efficiency, HCG makes it possible to build models of arbitrary sequence with prebuilt fragment libraries, given the limited four-letter alphabet of RNA sequences. For proteins, where sampling of full-length chains is feasible in principle, HCG was found to provide substantially larger coverage of configuration space than MD simulations on an ≈2 μs time scale.^43^

## 4 Conclusions

Single-stranded RNA appears prominently in many cellular regulation processes, e.g., in mRNA and its poly-A tail but also in loops and linkers. The structural modeling of a flexible nucleic acid with unpaired nucleobases poses formidable challenges. Here, we showed that hierarchical chain growth, previously introduced for disordered proteins,^43^ can be used to produce structural ensembles of ssRNA with atomic detail, starting from fragments sampled in MD simulations. The resulting structural ensembles feature highly diverse conformations (Figures 2 and S3), in good agreement with NMR experiments probing the local structure (Figure 5D). Also SAXS and FRET experiments probing the global structure are reproduced well. Overall, we found the HCG ensembles to agree with experiments about as well or better than MD simulations of full-length ssRNA (Figures 3–5, 7).

HCG relies on a number of simplifying assumptions. Most importantly, it assumes that the relevant local structure of disordered biopolymers is sampled properly in short fragments and that these fragments can be assembled into full length chains subject to only steric interactions. In particular, in its simplest form, HCG does not account for long-range electrostatic interactions. It is therefore remarkable that we obtained excellent agreement for rA_30_ SAXS data over a wide range of salt concentrations, in particular for 100–200 mM NaCl (Figure 4).

The computational efficiency of HCG makes it possible to construct large ensembles with diverse conformations, sampling also significant populations of rare but relevant conformations. This broad coverage of conformation space enables ensemble reweighting schemes to match a wide range of experiments within expected uncertainties (Figures 3–5, 7, S6, S7, S9–S13, S15A and B, and S17).

HCG is implemented as a Monte Carlo chain growth algorithm with a well-defined ensemble and partition function.^43^ Therefore, HCG can easily be combined with other Monte Carlo sampling techniques, e.g., to perform importance sampling as in the reweighted hierarchical chain growth (RHCG).^40^ In RHCG, one uses a fragment library that is refined against experimental data prior to fragment assembly to improve the sampling of local properties in the grown full-length ensemble. An exciting perspective is to adopt an RHCG-like sampling scheme to include information on interfragment interactions during chain growth or to grow loop structures. This task may be turned into a machine learning problem. Methods based on artificial intelligence (AI) have been proven to reliably predict tertiary structure of folded double-stranded and also single-stranded RNA.^30,71,75,76^ Query sequences that require modeling of both structured and disordered regions may be excellent targets for AI-guided applications of Monte Carlo techniques. We have shown that HCG is suited to model segments of mRNA that feature structured and unstructured regions (see Figure 6). HCG in combination with machine learning approaches could prove useful for modeling more complicated mRNA or long-noncoding RNA (lncRNA) with internal short disordered loops. To improve the grown structures, one can include experimentally derived information^40^ and information from secondary structure prediction tools. Fragment libraries for 3D structures of RNA secondary structure motifs^77^ can be used as input for HCG of more complex RNA folds. One could also use coarse-grained RNA simulation models^78^ to build fragment libraries for the assembly of large RNA structures.

HCG can also be combined with MD simulations to gain insight on inter- and intramolecular interactions and the dynamics of ssRNA. In previous work, we have shown that the conformations of IDPs sampled with HCG are well-suited as starting structures for parallel but independent MD simulations with atomic detail.^43^ Similarly, this could be done with the ssRNA conformations as modeled here, either the fully flexible single chains or molecules with structured and flexible regions. Starting from a multitude of reasonable initial structures will facilitate exploration of the relevant conformational space and dynamics.

## Data Availability

An implementation of BioEn specific for SAXS data that fits nuisance parameters globally for the ensemble average during the refinement was used, with the code available at https://github.com/bio-phys/SAXS_BioEn/. The RNA structure libraries (i.e., the MD fragment library as well as exemplary structures from the HCG ensembles discussed in this work) are available at https://zenodo.org/record/8369324. The HCG code to assemble ssRNA to the hierarchical chain growth is available at the GitHub repository https://github.com/bio-phys/hierarchical-chain-growth/.

## Supporting Information Available

Supporting table of SAXS fit parameters. Supporting figures of Cluster analysis of the MD trajectory of a rA_4_ fragment; sequence of the SARS-CoV-2 5′ UTR as modeled here; snapshots of rA_19_ models from HCG with attached FRET labels; stacking analysis of poly A and poly U 30mers from HCG; backbone dihedral angle distributions sampled in rA_4_ MD trajectories and rA_19_ HCG models; BioEn reweighting of rA_30_ HCG ensemble against SAXS data at different salt concentrations; BioEn reweighting of rU_30_ HCG ensemble against experimental SAXS data; distribution of the radius of gyration for HCG rA_30_ ensembles from different fragment sequences; refinement of rA_30_ ensemble against SAXS data at 0.1, 0.02, 0.2, 0.4, and 0.6 M NaCl; comparison of SAXS intensities calculated for rA_30_ and rU_30_ against experiments for rA_30_ at different salt conditions; BioEn reweighting of rUCAAUC HCG ensemble against experimental SAXS data; CDF of pairwise RMSDs in (RE)MD ensembles and HCG ensembles of ssRNA polymers with different lengths; BioEn reweighting of rA_19_ HCG ensemble against experimental FRET data; comparison of HCG to earlier MD simulations of full-length rU_40_ on Anton (PDF)
