# Supplementary material for: Hierarchical Assembly of Single-Stranded RNA
Source: J Chem Theory Comput. 2024 Feb 16;20(5):2246–60. doi: 10.1021/acs.jctc.3c01049 (PMC10938505; doi:10.1021/acs.jctc.3c01049)
Supplement: Supplementary file 1 — ct3c01049_si_001.pdf [file ct3c01049_si_001.pdf]

Supporting Information: Hierarchical Assembly of Single-Stranded RNA

Lisa M. Pietrek,† Lukas S. Stelzl,‡,¶,§ and Gerhard Hummer*,†,‖

†*Department of Theoretical Biophysics, Max Planck Institute of Biophysics, Max-von-Laue-Straße 3, 60438 Frankfurt am Main, Germany*

‡*Faculty of Biology, Johannes Gutenberg University Mainz, Gresemundweg 2, 55128 Mainz, Germany*

¶*KOMET 1, Institute of Physics, Johannes Gutenberg University Mainz, 55099 Mainz, Germany*

§*Institute of Molecular Biology (IMB), 55128 Mainz, Germany*

‖*Institute for Biophysics, Goethe University Frankfurt, Max-von-Laue-Straße 9, 60438 Frankfurt am Main, Germany*

E-mail: gerhard.hummer@biophys.mpg.de

Contents

Supporting Table S1 **S-2**

Supporting Figures S1–S18 **S-3**

References **S-17**

Supporting Table S1

Table S1: Least-square fitting of SAXS intensities. In least-square fits of $I(q) = a\, I_{\mathrm{sim}}(q) + b$, the intensity scaling factor a and the constant background correction factor b were adjusted. Results are listed for a fit for the scattering profiles of rU_{30} from experiment (100 mM NaCl) and from HCG (150 mM NaCl) to the experimental scattering profiles of rA_{30} at different salt concentrations. The experimental profiles were previously reported by Plumridge et al.[S1]

**SAXS scattering profiles**	**reduced** χ^2	**a**	**b**
rU_{30} (experiment) to rA_{30} (100 mM NaCl)	0.9	8.1×10^{-1}	-9.2×10^{-2}
rU_{30} (HCG) to rA_{30} (20 mM NaCl)	7.0	4.5×10^{-6}	6.2×10^{-1}
rU_{30} (HCG) to rA_{30} (100 mM NaCl)	3.7	5.4×10^{-6}	5.5×10^{-1}
rU_{30} (HCG) to rA_{30} (200 mM NaCl)	3.8	5.7×10^{-6}	5.1×10^{-1}
rU_{30} (HCG) to rA_{30} (400 mM NaCl)	3.7	6.9×10^{-6}	5.1×10^{-1}
rU_{30} (HCG) to rA_{30} (600 mM NaCl)	1.6	3.3×10^{-6}	3.8×10^{-1}

Supporting Figures S1–S18

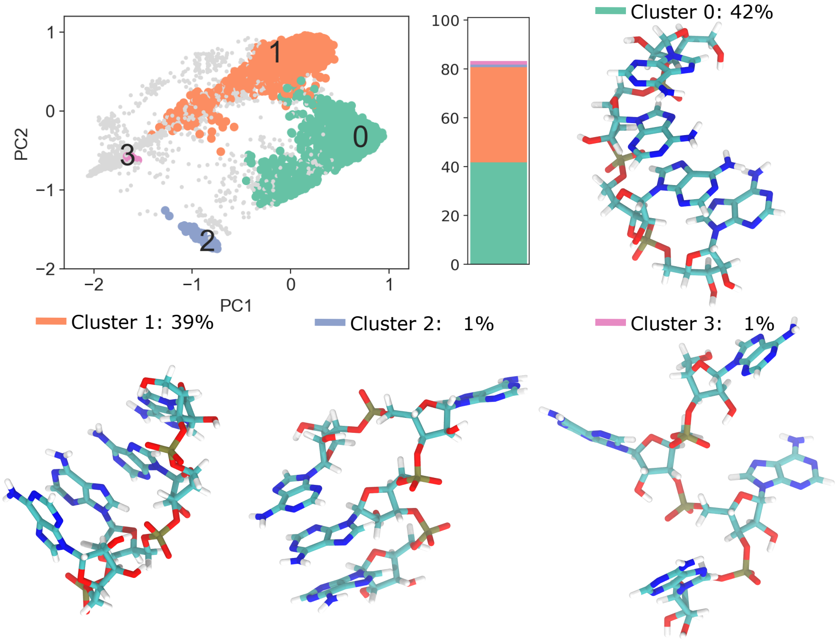

Figure S1: Cluster analysis of the MD trajectory of rA_4. (A) PCA of the g-vectors of 10000 conformations sampled in 100 ns of replica exchange molecular dynamics (REMD) simulations. The conformations are assigned to six different clusters. The four most populated clusters are highlighted in color and a representative structure for each cluster is shown (B-E). The cluster analysis was performed using the Barnaba package.[S2]

SL1

1 AUUAAGGGUU UAUACCUUCC CAGGUAACAA ACCCAACCAA CUUUCGGAUC

SL2+SL3

51 UCUUGUAGAU CUGUUCUCUA AACGAACGAA CUUUAAAAUC UGGGUGUGGC

SL4

101 UGUCACUCGG CUGCAUGCUU AGUGCACUCA CGCCCAGUAU AAUUAAUAAC

151 UAAUUUUACU GUCGUUGACA GGACACGAGU AACUCGUCUA UCUUCUGCAG

SL5a

201 GGCUGCUUAC GGUUUCGUCC GUGUUGCAGC CCC

Figure S2: Sequence of the SARS-CoV-2 5′ UTR as modelled here. Base paired stem-loop (SL) regions as sampled with MD, published previously,[S3] are highlighted in red; Single-stranded regions modelled with HCG are highlighted in blue. Nucleobases 162-200 are part of SL5, i.e., are predicted to be structured. However, in our example this region is treated as unstructured and modelled with HCG (underlined in red). Sequence information was taken from ref S3.

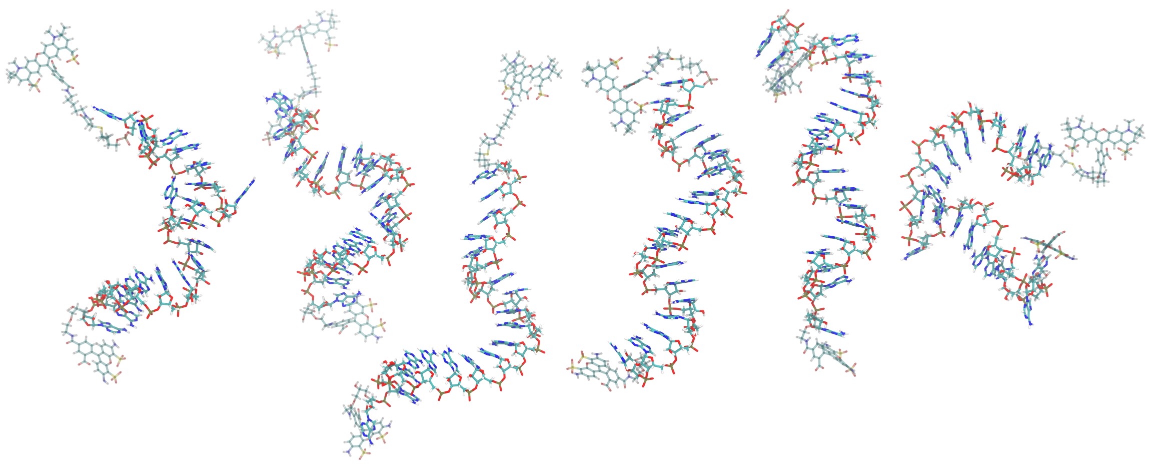

Figure S3: Snapshots of rA_{19} models from HCG with attached FRET labels Alexa Fluor 594 and Alexa Fluor 488 at the 5′ and 3′ ends, respectively.

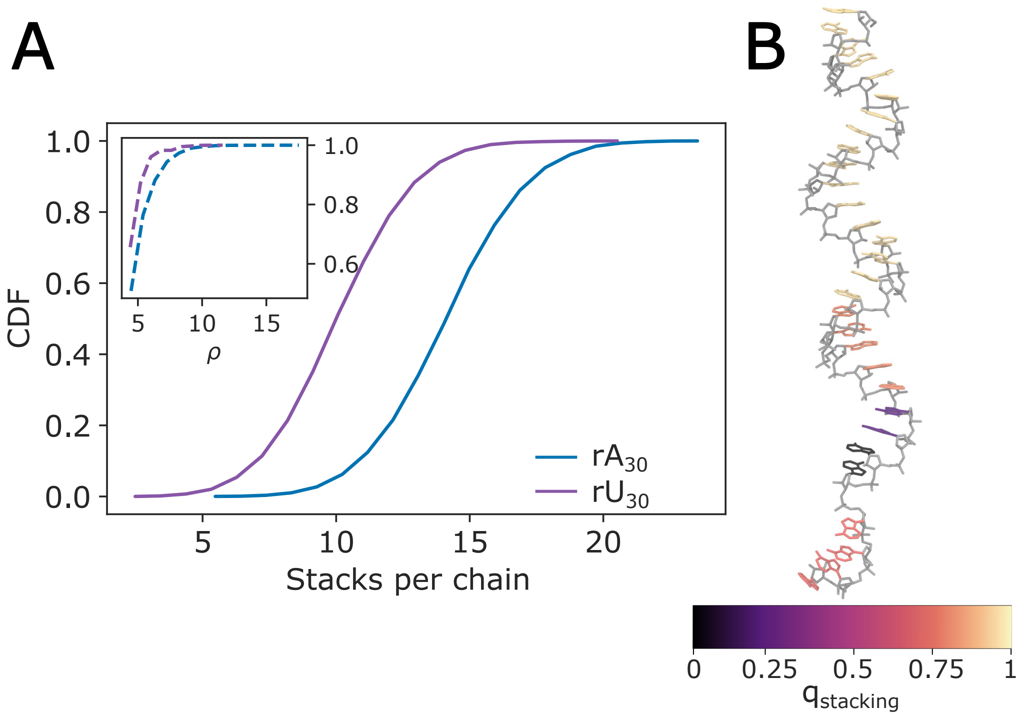

Figure S4: Stacking analysis of rA_{30} and rU_{30} ensembles grown with HCG. (A) Cumulative distribution function (CDF) of the number of base-pair stacks per chain for rA_{30} in blue and rU_{30} in violet. One stack is formed by two nucleobases. The inset shows the CDF of nucleobases involved in consecutive stacks (ρ) for both polymers. We define a consecutive stack as four or more stacked nucleobases. (B) A render of rA_{30} with nucleobases colored according to the stacking. Hydrogen atoms are omitted for clarity. The nucleic acid backbone and the sugar moiety are shown in gray. The stacking analysis was performed using the Barnaba package.[S2]

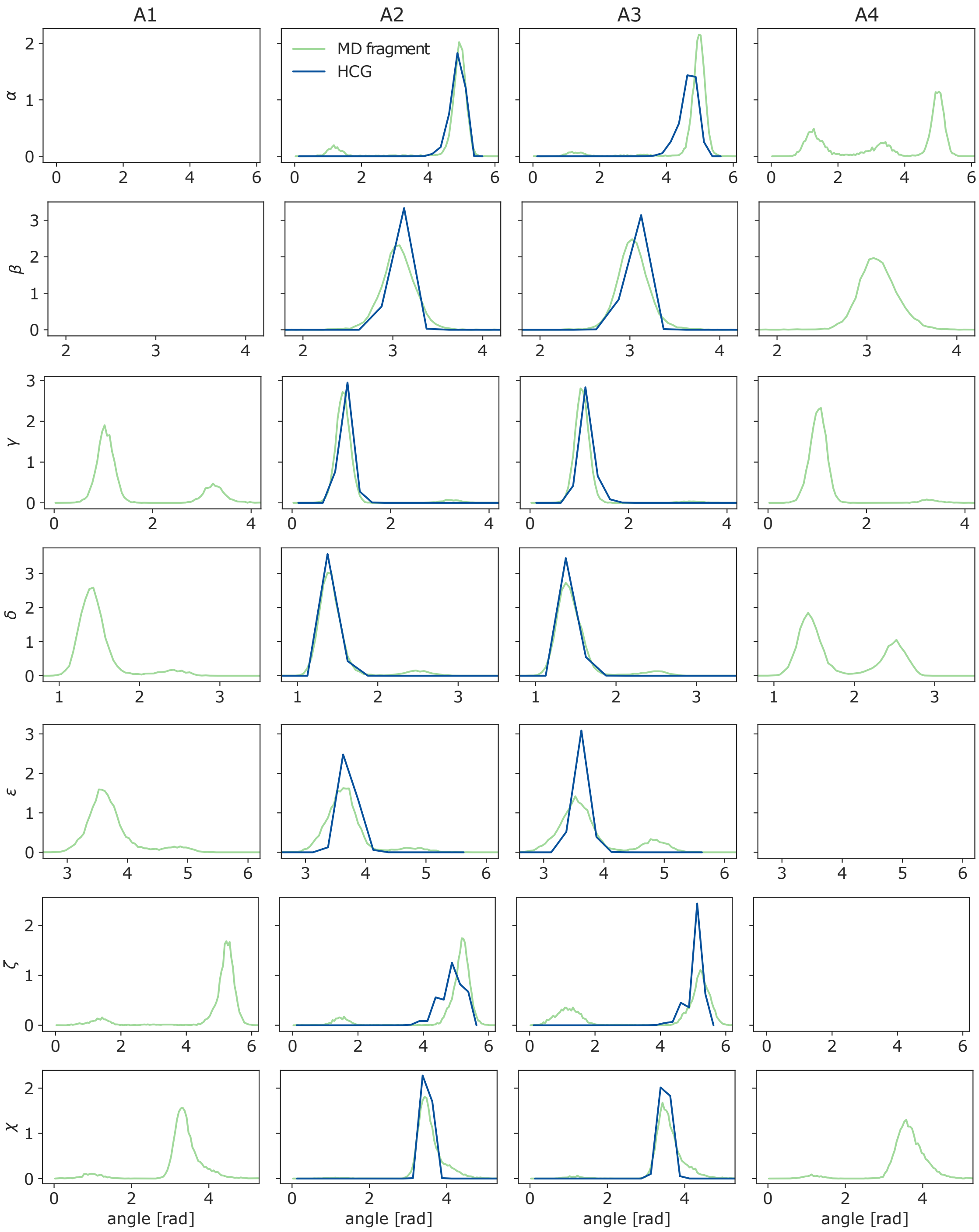

Figure S5: Distributions of backbone dihedral angles of the adenosine nucleotides as sampled in the MD fragment and the assembled rA_{19} homopolymer from HCG. (Top to bottom) Distributions of the α, β, γ, δ, ϵ, ζ, and χ angles of nucleotides A1-A4 (left to right). Results for the rA_4 tetramer fragment sampled in 100 ns REMD simulation are shown in light green. The distributions of the backbone dihedral angles of the A2 and A3 nucleotides after being assembled into rA_{19} with HCG are shown in blue. For rA_{19}, the distributions of the respective dihedral angles were averaged over all residues in the second (A2) and third (A3) position of the respective rA_4 fragment.

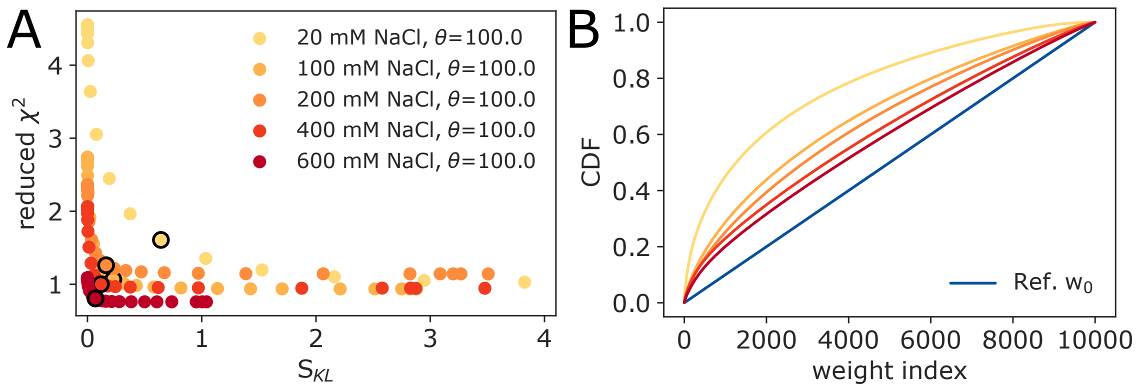

Figure S6: Assessment of the BioEn reweighting of the rA_{30} structural HCG ensemble against SAXS measurements carried out at different NaCl concentrations. (A) L-curve analysis for the BioEn refinement results for NaCl concentrations 20, 100, 200, 400, and 600 mM. (B) Cumulative distribution of rank ordered weights. Uniform reference weights w_0 are shown in blue, refined weights from BioEn for $\theta = 100$ are shown in colors from dark red to yellow for the different salt concentrations. Color scheme as indicated in the legend of panel A.

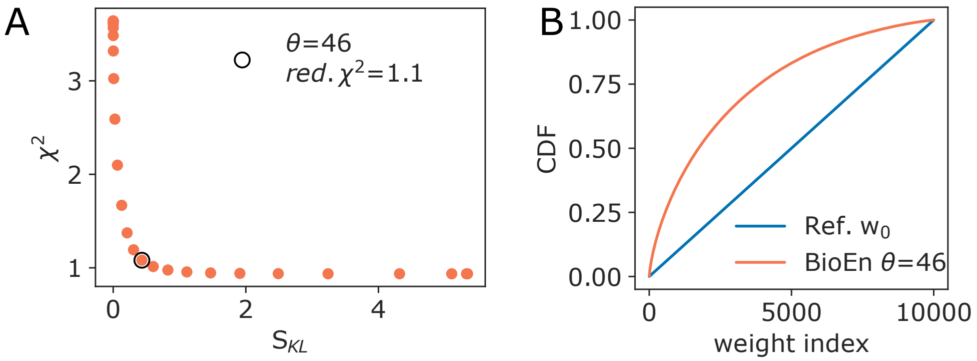

Figure S7: Ensemble refinement of a rU_{30} HCG ensemble against experimental SAXS data. The experimental profile was recorded at 100 mM NaCl by Plumridge et al.[S1] (A) L-curve analysis with reduced χ^2 plotted against S_{KL} as a result of the BioEn reweighting. (B) Cumulative distribution of rank ordered weights for the uniform reference weights w_0 and refined weights for $\theta = 46$ (blue and orange, respectively).

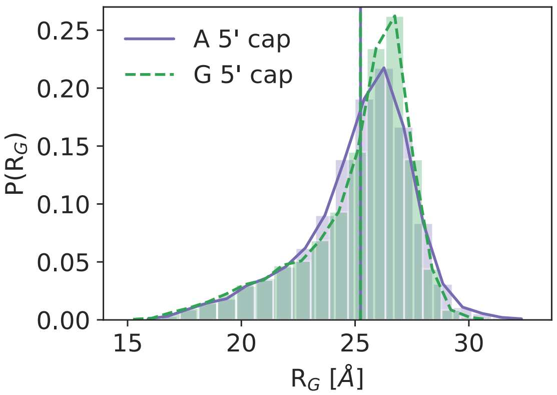

Figure S8: Distribution of the radius of gyration R_G for rA_{30} HCG ensembles assembled from rA_4 (purple) and rGA_3 fragment libraries (green), respectively. Vertical lines indicate the RMS R_G values.

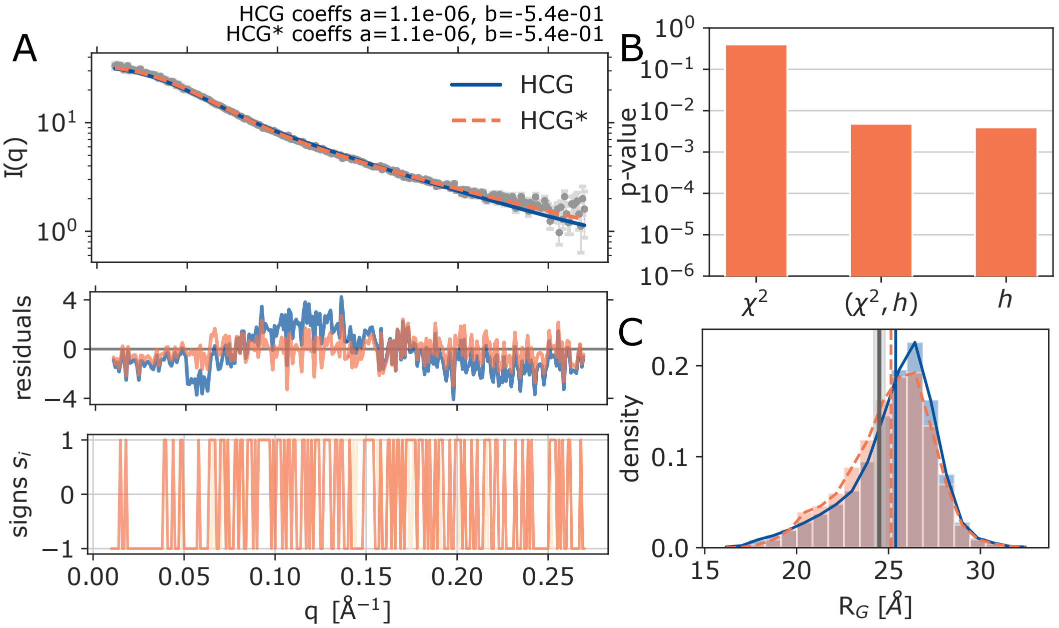

Figure S9: Ensemble refinement of a rA_{30} HCG ensemble against experimental SAXS data recorded at 100 mM NaCl by Plumridge et al.[S1] (A) Top: Scattering profiles for experiment, unrefined HCG, refined HCG* ensemble (gray, blue, and orange) with the respective fitting parameters (intensity scale factor a and the background correction constant b). Middle: Residuals for the SAXS data. Bottom: Signs analysis of the residuals. (B) P-values of the refined ensemble for reduced χ^2, combined reduced χ^2 and h, and h as calculated by the hplusminus analysis package.[S4] (C) Distribution of R_G sampled in HCG (without refinement) and HCG* (after refinement of the weights to match the SAXS data), and their averages (blue and dotted orange vertical lines). The experimental value determined at 100 mM NaCl is shown as dark gray vertical line with the error range highlighted as light gray shaded area.

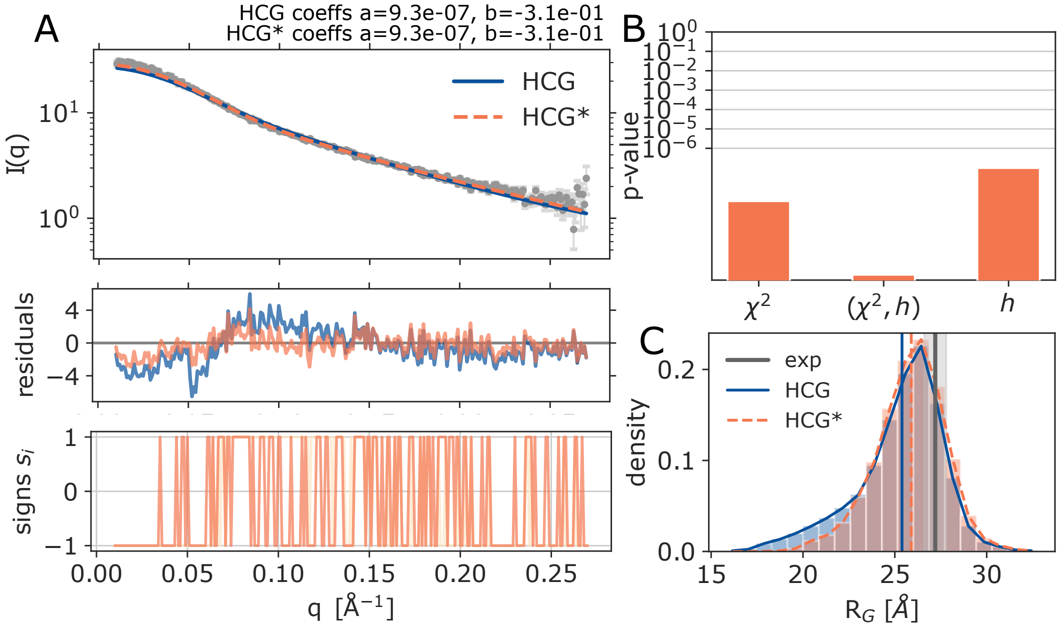

Figure S10: Ensemble refinement of a rA_{30} HCG ensemble against experimental SAXS data recorded at 20 mM NaCl by Plumridge et al.[S1] (A) Top: Scattering profiles for experiment, unrefined HCG, refined HCG* ensemble (gray, blue, and orange) with the respective fitting parameters (intensity scale factor a and the background correction constant b). Middle: Residuals for the SAXS data. Bottom: Signs analysis of the residuals. (B) P-values of the refined ensemble for reduced χ^2, combined reduced χ^2 and h, and h as calculated by the hplusminus analysis package.[S4] (C) Distribution of R_G sampled in HCG (without refinement) and HCG* (after refinement of the weights to match the SAXS data), and their averages (blue and dotted orange vertical lines). The experimental value determined at 20 mM NaCl is shown as dark gray vertical line with the error range highlighted as light gray shaded area.

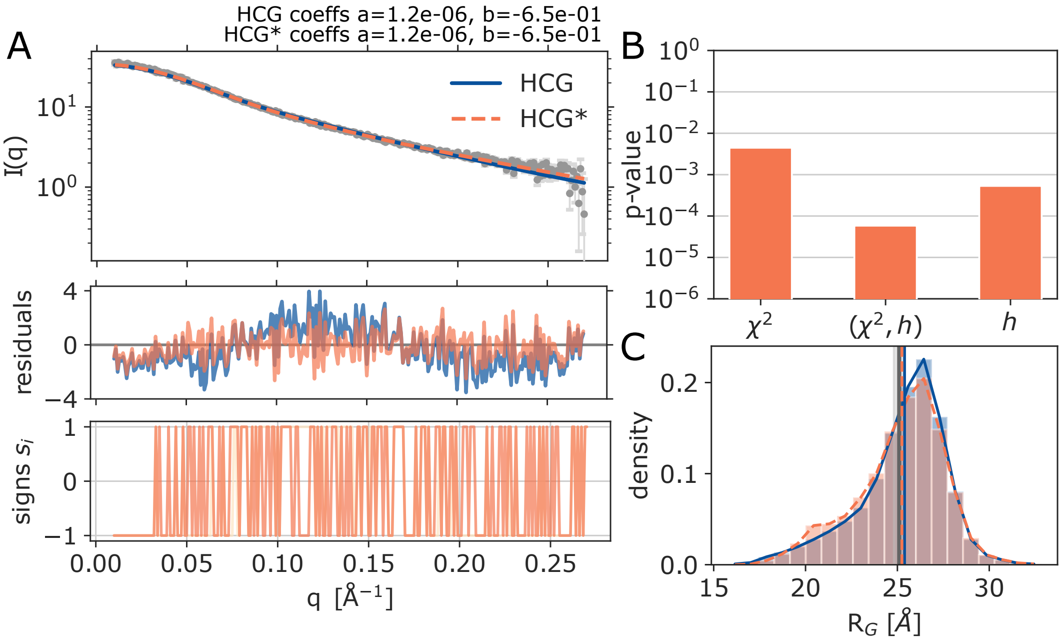

Figure S11: Ensemble refinement of a rA_{30} HCG ensemble against experimental SAXS data recorded at 200 mM NaCl by Plumridge et al.[S1] (A) Top: Scattering profiles for experiment, unrefined HCG, refined HCG* ensemble (gray, blue, and orange) with the respective fitting parameters (intensity scale factor a and the background correction constant b). Middle: Residuals for the SAXS data. Bottom: Signs analysis of the residuals. (B) P-values of the refined ensemble for reduced χ^2, combined reduced χ^2 and h, and h as calculated by the hplusminus analysis package.[S4] (C) Distribution of R_G sampled in HCG (without refinement) and HCG* (after refinement of the weights to match the SAXS data), and their averages (blue and dotted orange vertical lines). The experimental value determined at 200 mM NaCl is shown as dark gray vertical line with the error range highlighted as light gray shaded area.

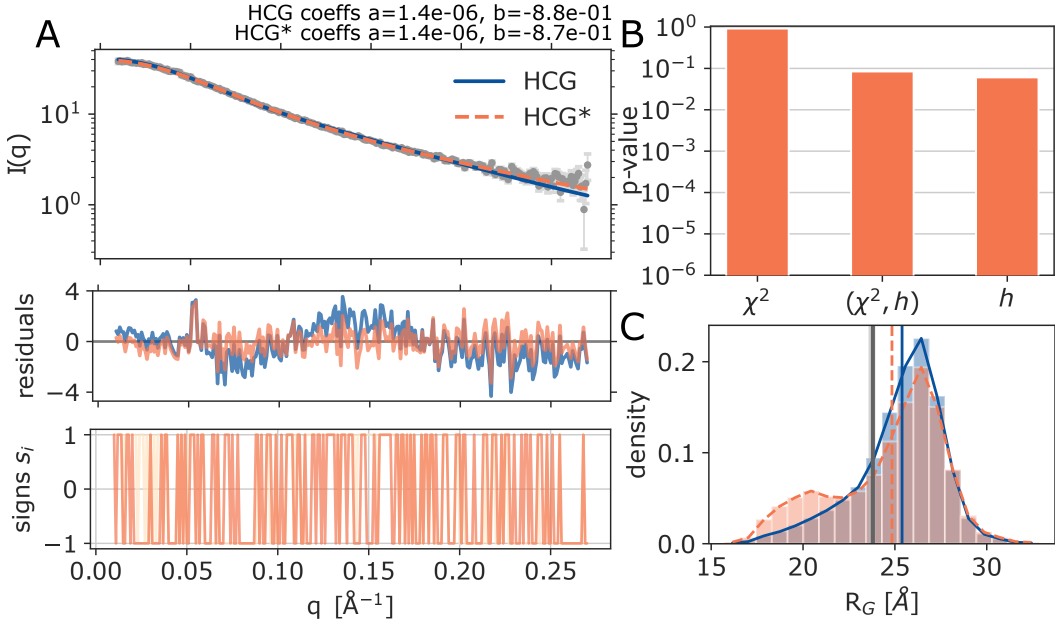

Figure S12: Ensemble refinement of a rA_{30} HCG ensemble against experimental SAXS data recorded at 400 mM NaCl by Plumridge et al.[S1] (A) Top: Scattering profiles for experiment, unrefined HCG, refined HCG* ensemble (gray, blue, and orange) with the respective fitting parameters (intensity scale factor a and the background correction constant b). Middle: Residuals for the SAXS data. Bottom: Signs analysis of the residuals. (B) P-values of the refined ensemble for reduced χ^2, combined reduced χ^2 and h, and h as calculated by the hplusminus analysis package.[S4] (C) Distribution of R_G sampled in HCG (without refinement) and HCG* (after refinement of the weights to match the SAXS data), and their averages (blue and dotted orange vertical lines). The experimental value determined at 400 mM NaCl is shown as dark gray vertical line with the error range highlighted as light gray shaded area.

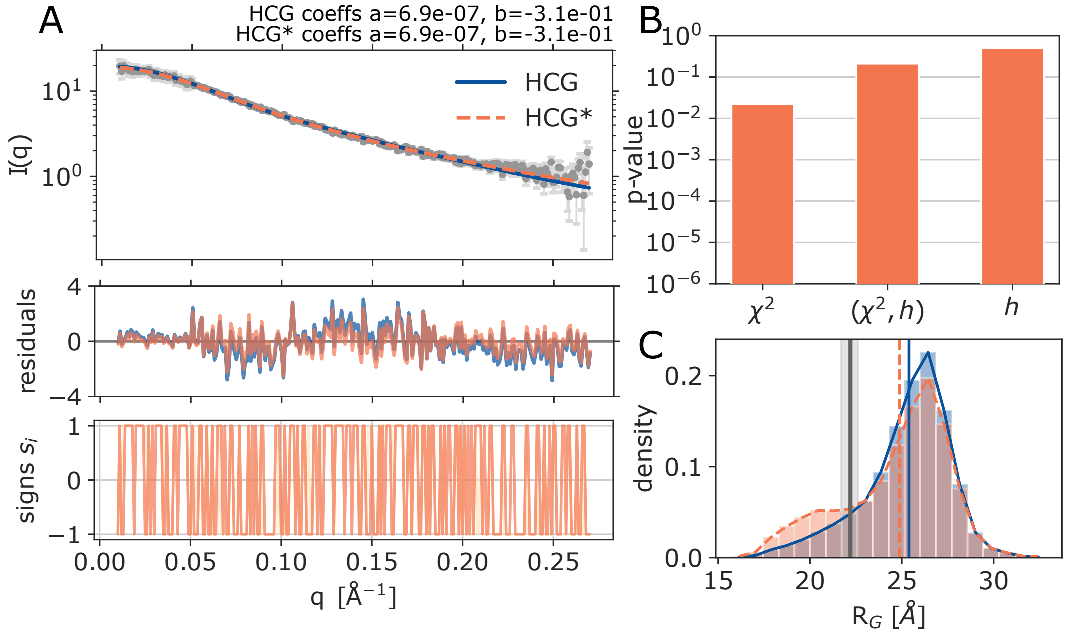

Figure S13: Ensemble refinement of a rA_{30} HCG ensemble against experimental SAXS data recorded at 600 mM NaCl by Plumridge et al.[S1] (A) Top: Scattering profiles for experiment, unrefined HCG, refined HCG* ensemble (gray, blue, and orange) with the respective fitting parameters (intensity scale factor a and the background correction constant b). Middle: Residuals for the SAXS data. Bottom: Signs analysis of the residuals. (B) P-values of the refined ensemble for reduced χ^2, combined reduced χ^2 and h, and h as calculated by the hplusminus analysis package.[S4] (C) Distribution of R_G sampled in HCG (without refinement) and HCG* (after refinement of the weights to match the SAXS data), and their averages (blue and dotted orange vertical lines). The experimental value determined at 600 mM NaCl is shown as dark gray vertical line with the error range highlighted as light gray shaded area.

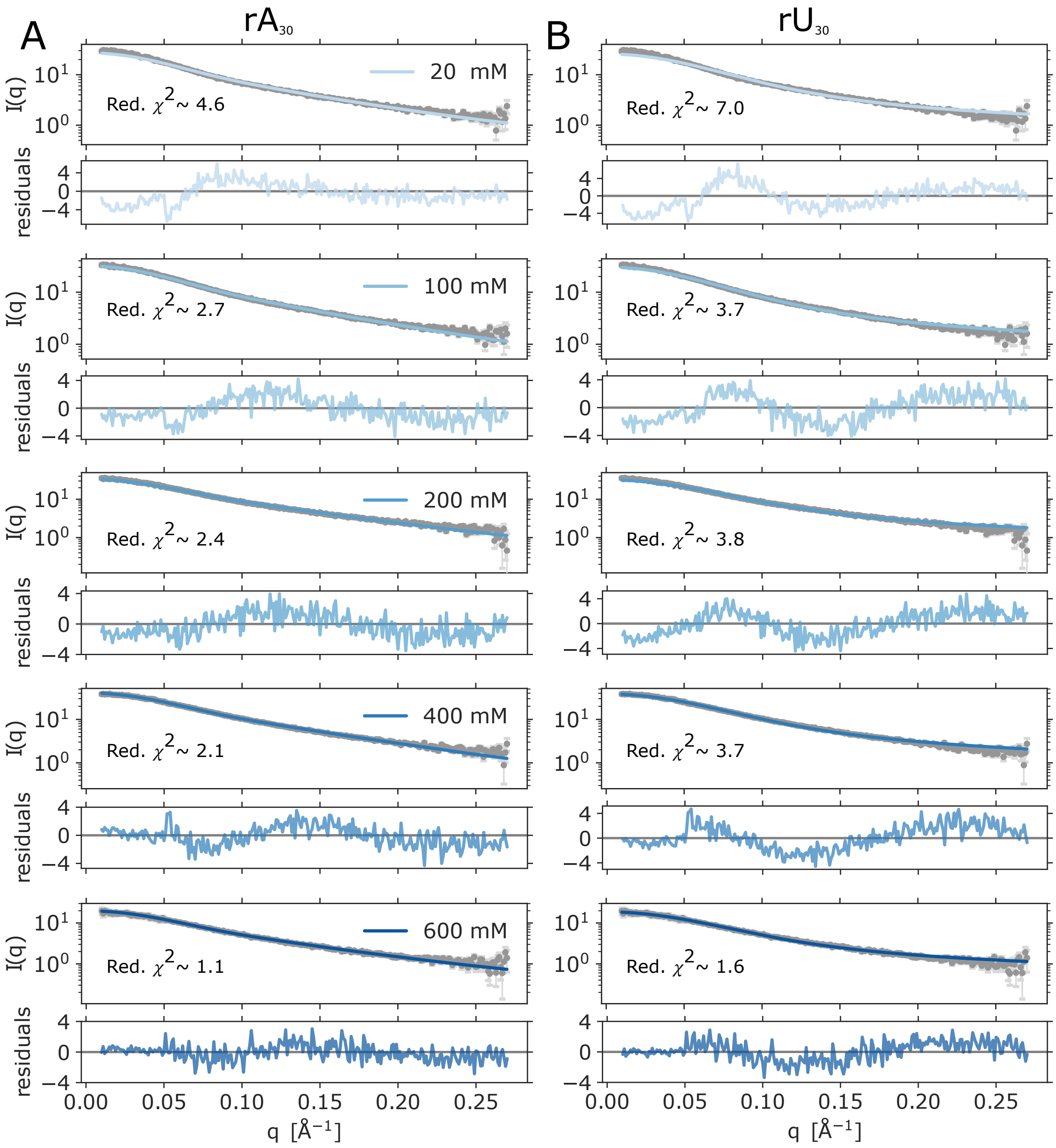

Figure S14: Comparison of scattering profiles computed for rA_{30} and rU_{30} HCG ensembles to experimental salt-dependent scattering profiles of rA_{30} recorded by Plumridge et al.[S1] Scattering profiles (top) and residuals (bottom) of rA_{30} and rU_{30} HCG ensembles after least-square fitting of a and b to experimental profiles recorded at 20, 100, 200, 400, and 600 mM NaCl (top to bottom). The fitted intensity scale factors a and background correction constants b for rU_{30} are listed in Table S1 and for rA_{30} in Figures S10A-S13A, respectively.

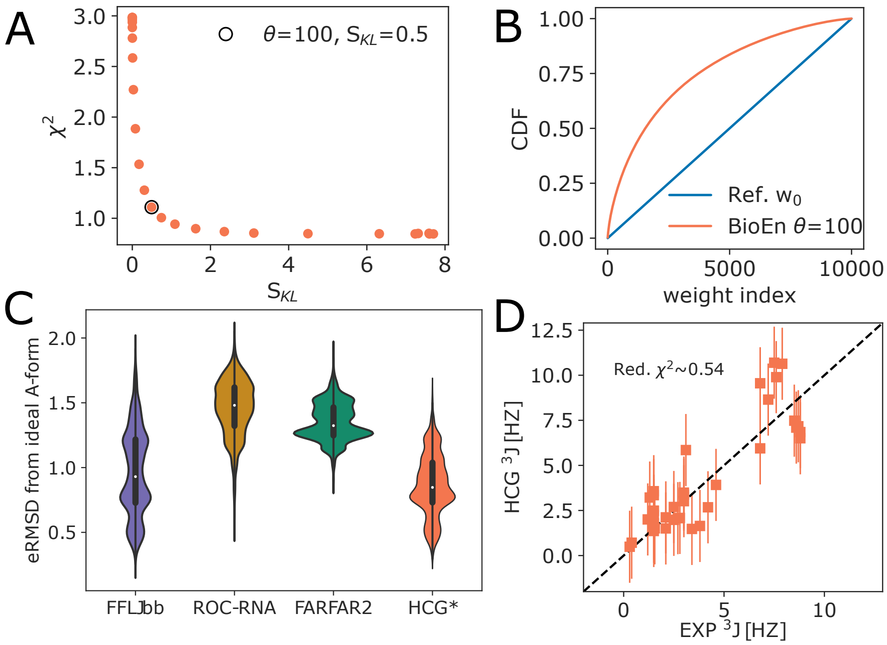

Figure S15: Structural ensembles of heteropolymeric UCAAUC from HCG reweighted against the experimental scattering profile.[S5] (A) L-curve analysis of the BioEn refinement. We chose refined weights for $\theta = 100$ for further analysis of properties shown here. (B) Cumulative distribution of rank-ordered weights. Uniform reference weights are shown in blue, refined weights for $\theta = 100$ in orange. (C) Distribution of the eRMSD to ideal A-form. (D) Correlation plot of ^3J-couplings of experimentally measured[S6] and calculated values for the refined HCG* ensemble. Vertical bars indicate the estimated uncertainty of ± 2 Hz (one standard error) in calculating the ^3J-couplings from RNA structures using approximate Karplus relations.[S6,S7]

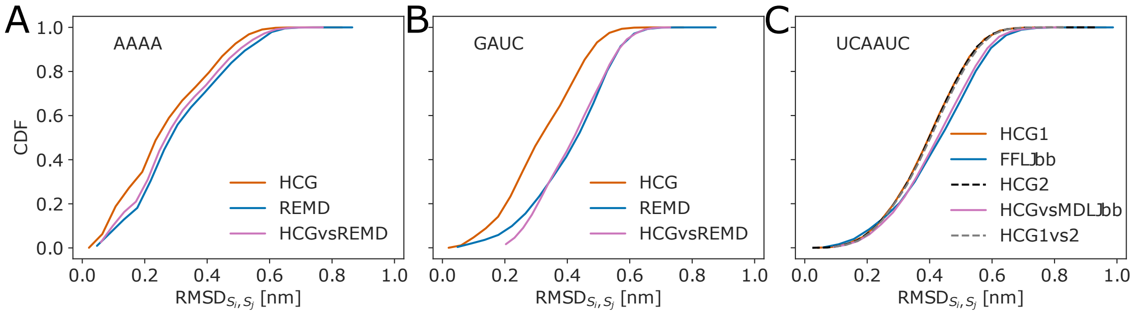

Figure S16: Cumulative distribution of pairwise RMSDs in (RE)MD ensembles and HCG ensembles of ssRNA polymers with different lengths. CDFs are shown for the pairwise RMSD within (RE)MD simulations (blue), within HCG ensembles (dark orange and dotted black), and between random structures S_i and S_j in different ensembles (rose and gray). Pairwise RMSDs were calculated using all heavy atoms within a polymer. Each polymer ensemble sampled with either (RE)MD or HCG contained 10000 structures. (A) rAAAA and (B) rGAUC tetramer, REMD run for 100 ns with the DESRES force field combined with the TIP4P-d water model. (C) rUCAAUC hexamer with the MD trajectory run for 1 μs with the LJbb force field by Bergonzo et al.[S5] Here, the RMSD between two independent HCG ensembles of rUCAAUC is also shown (dotted gray).

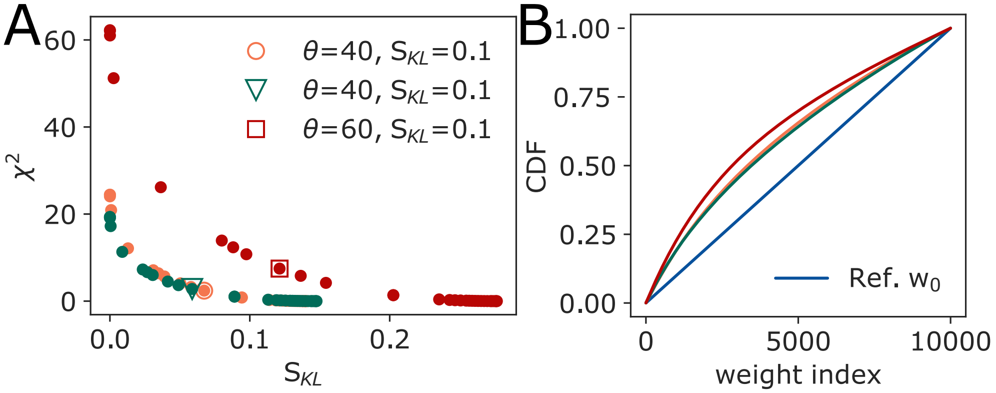

Figure S17: Assessment of the refinement of the structural HCG ensemble of rA_{19} against single-molecule FRET experiments. We reweighted the distribution of FRET efficiencies calculated from a HCG ensemble with 10000 chains of rA_{19} with mapped dyes calculated with model 1 (dynamic dye orientations, orange), model 2 (dynamic dyes, dark green), or model 3 (static dyes,[S8] dark red) against the experimental mean efficiency $\langle E \rangle$ using BioEn.[S9] (A) L-curve analysis of the results from reweighting. We found $S_{\mathrm{KL}} < 0.1$ and $\chi^2 \approx 2.7$ for a set of weights for $\theta = 40$ for model 1 (orange empty circle). For model 2 we chose weights for $\theta = 40$ with $S_{\mathrm{KL}} < 0.1$ and $\chi^2 \approx 7.8$ (dark green empty triangle). For model 3 we chose a set of weights for $\theta = 60$ with $S_{\mathrm{KL}} \approx 1.4$ and $\chi^2 \approx 8$ (dark red empty square). (B) Cumulative distribution of rank ordered weights for the respective θ. Color scheme as indicated in panel A.

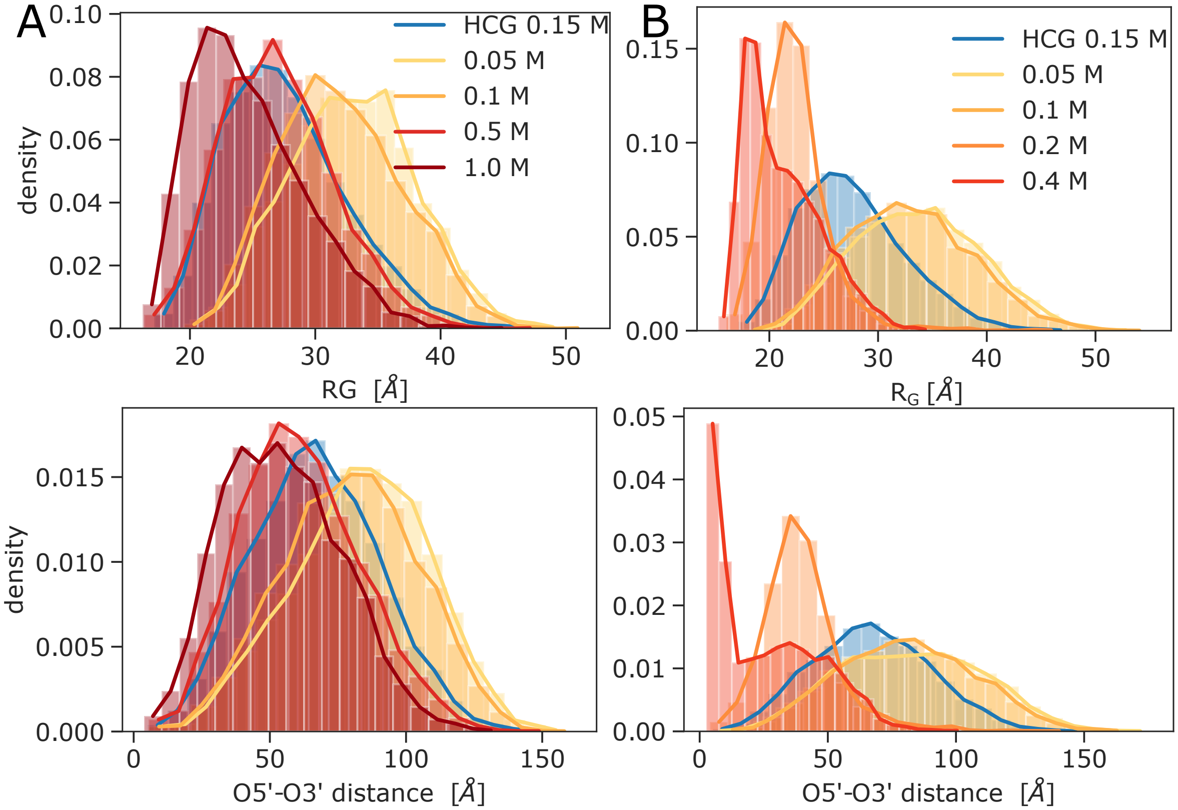

Figure S18: Distributions of the radius of gyration (R_G, top) and O5′-O3′ distance (bottom) of rU_{40} from HCG and MD simulations using the Anton supercomputer.[S10,S11] The HCG ensemble contained 10000 rU_{40} chains assembled from MD fragments sampled in an aqueous solution of 0.15 M NaCl with the DESRES[S10] (=DES-Amber09) force field (blue). For comparison, results are shown for earlier MD simulations[S10,S11] of full-length rU_{40} (A) in solutions of 0.05, 0.1, 0.5, and 1.0 M NaCl with the DES-Amber09 force field (yellow and light orange) and (B) in solutions of 0.05, 0.1, 0.2, and 0.4 M NaCl with the DES-Amber09_pe3.20 force field (yellow to dark red).

References

(S1) Plumridge, A.; Andresen, K.; Pollack, L. Visualizing disordered single-stranded RNA: connecting sequence, structure, and electrostatics. *J. Am. Chem. Soc.* **2020**, *142*, 109–119.

(S2) Bottaro, S.; Bussi, G.; Pinamonti, G.; Reiber, S.; Boomsma, W.; Lindorff-Larsen, K. Barnaba: software for analysis of nucleic acid structures and trajectories. *RNA* **2019**, *25*, 219–231.

(S3) Bottaro, S.; Bussi, G.; Lindorff-Larsen, K. Conformational ensembles of noncoding elements in the SARS-CoV-2 genome from molecular dynamics simulations. *J. Am. Chem. Soc.* **2021**, *143*, 8333–8343.

(S4) Köfinger, J.; Hummer, G.; Köfinger, J. Powerful statistical tests for ordered data. *ChemRxiv* **2021**, DOI:10.26434/chemrxiv-2021-mdt29-v3.

(S5) Bergonzo, C.; Grishaev, A.; Bottaro, S. Conformational heterogeneity of UCAAUC RNA oligonucleotide from molecular dynamics simulations, SAXS, and NMR experiments. *RNA* **2022**, *28*, 937–946.

(S6) Zhao, J.; Kennedy, S. D.; Berger, K. D.; Turner, D. H. Nuclear magnetic resonance of single-stranded RNAs and DNAs of CAAU and UCAAUC as benchmarks for molecular dynamics simulations. *J. Chem. Theory Comput.* **2020**, *16*, 1968–1984.

(S7) Bottaro, S.; Bussi, G.; Kennedy, S. D.; Turner, D. H.; Lindorff-Larsen, K. Conformational ensembles of RNA oligonucleotides from integrating NMR and molecular simulations. *Sci. Adv.* **2018**, *4*.

(S8) Hummer, G.; Szabo, A. Dynamics of the orientational factor in fluorescence resonance energy transfer. *J. Phys. Chem. B* **2017**, *121*, 3331–3339.

(S9) Hummer, G.; Köfinger, J. Bayesian ensemble refinement by replica simulations and reweighting. *J. Chem. Phys.* **2015**, *143*.

(S10) Tan, D.; Piana, S.; Dirks, R. M.; Shaw, D. E. RNA force field with accuracy comparable to state-of-the-art protein force fields. *Proc. Natl. Acad. Sci. USA* **2018**, *115*, E1346–E1355.

(S11) Tucker, M. R.; Piana, S.; Tan, D.; LeVine, M. V.; Shaw, D. E. Development of force field parameters for the simulation of single- and double-stranded DNA molecules and DNA–protein complexes. *J. Phys. Chem. B* **2022**, *126*, 4442–4457.
